# The Architecture of a Prototypical Bacterial Signaling Circuit Enables a Single Point Mutation to Confer Novel Network Properties

**DOI:** 10.1371/journal.pgen.1003706

**Published:** 2013-08-22

**Authors:** Sri Ram, Mark Goulian

**Affiliations:** Department of Biology, University of Pennsylvania, Philadelphia, Pennsylvania, United States of America; Baylor College of Medicine, United States of America

## Abstract

Even a single mutation can cause a marked change in a protein's properties. When the mutant protein functions within a network, complex phenotypes may emerge that are not intrinsic properties of the protein itself. Network architectures that enable such dramatic changes in function from a few mutations remain relatively uncharacterized. We describe a remarkable example of this versatility in the well-studied PhoQ/PhoP bacterial signaling network, which has an architecture found in many two-component systems. We found that a single point mutation that abolishes the phosphatase activity of the sensor kinase PhoQ results in a striking change in phenotype. The mutant responds to stimulus in a bistable manner, as opposed to the wild-type, which has a graded response. Mutant cells in on and off states have different morphologies, and their state is inherited over many generations. Interestingly, external conditions that repress signaling in the wild-type drive the mutant to the on state. Mathematical modeling and experiments suggest that the bistability depends on positive autoregulation of the two key proteins in the circuit, PhoP and PhoQ. The qualitatively different characteristics of the mutant come at a substantial fitness cost. Relative to the off state, the on state has a lower fitness in stationary phase cultures in rich medium (LB). However, due to the high inheritance of the on state, a population of on cells can be epigenetically trapped in a low-fitness state. Our results demonstrate the remarkable versatility of the prototypical two-component signaling architecture and highlight the tradeoffs in the particular case of the PhoQ/PhoP system.

## Author Summary

A mutation can cause significant changes to a protein's function. Since proteins often act together in genetic circuits to control various cellular processes, mutant proteins can lead to unexpected consequences for system-level behavior. In this study, we describe a remarkable example of this phenomenon in a mutant of a well-studied bacterial circuit. PhoQ and PhoP are the primary regulatory proteins in a circuit that responds to low magnesium. The wild-type (unmutated) network responds to environmental signals in an analog or graded manner. In contrast, the mutant responds to signals in an OFF-or-ON or digital fashion. Moreover, the distribution of OFF and ON cells is strongly influenced by how cells were cultured in the past. These remarkable changes can be traced to features of the wiring diagram of the PhoQ/PhoP circuit. Since these features are shared among a broad class of bacterial signaling circuits, we suggest that other circuits may show similar remarkable properties when mutated.

## Introduction

A few mutations can lead to significant changes in a protein's functional properties. Examples include mutations that change the absorption and emission spectra of a fluorescent protein [1], the substrate specificity of an enzyme [2], or the allosteric control of a transcription factor [3]. In all of these examples, the change in phenotype can be directly traced to modifications in intrinsic properties of the protein. However, networks of interacting proteins can have system-level characteristics that bear a complex relationship to the intrinsic properties of the component molecules [4]. This complexity makes some network architectures inherently versatile, with different networks that share the same architecture exhibiting qualitatively different system-level behavior [5]. It remains a challenge to identify aspects of network architectures that promote versatility and permit novel properties to emerge by a few mutations to network components.

In this study, we demonstrate the versatility of the *E. coli* PhoQ/PhoP system. We show that a single point mutation in the histidine kinase PhoQ produces a striking change in the properties of the circuit. The PhoQ/PhoP system, which has an architecture found in many bacterial two-component signaling systems [6], responds to a variety of environmental conditions such as low Mg^2+^ [7], low pH [8], and the presence of cationic antimicrobial peptides [9], and controls transcription of a large set of genes [10]. The histidine kinase PhoQ senses these signals and modulates the phosphorylation level of the response regulator PhoP (PhoP-P), which functions as a transcription factor. PhoQ autophosphorylates and then transfers the phosphoryl group to PhoP, but also acts as a phosphatase, catalyzing PhoP-P dephosphorylation [11]. This bifunctional design, which is shared among many two-component systems, affects various properties of the system, including buffering the input-output relationship of the system to changes in histidine kinase and response regulator concentrations, and suppression of cross-talk [12]–[15]. The PhoQ/PhoP system is also autoregulated, that is, transcription of the *phoPphoQ* operon is activated by PhoP-P. Autoregulation is another common feature of many two-component systems [6] and is a mechanism for ultrasensitive response to stimulus without the need for cooperativity [16] as well as “learning” behaviors where prior exposure to stimulus improves response times to subsequent stimulating conditions [17], [18]. In the case of the PhoQ/PhoP system, autoregulation improves the dynamic range of the network output at high stimulus [13] and also gives rise to a surge in transcription upon activation [19].

The wild-type PhoQ/PhoP system responds to external stimulus in a graded rather than an all-or-none manner, with increasing stimulus (lower Mg^2+^ concentration) resulting in a higher mean response [13]. Moreover, the response of a population of cells is unimodal, with the PhoP-P levels of individual cells (inferred from transcriptional reporters) clustered around the population mean [13]. In this study, we show that a single point mutation that abolishes phosphatase activity in PhoQ produces a dramatic change in phenotype. The mutation, which produces a T281R substitution in PhoQ, results in bistability with mutants persisting in morphologically distinct OFF and ON states for many generations. We find that the architectural features of the network that allow the mutant to exhibit bistability are shared among many two-component systems. For PhoQ (T281R) mutants, however, the bistable phenotype comes at a fitness cost. We find that a population of cells can be epigenetically trapped in a low-fitness state.

## Results

### Bimodal Phenotype and Phenotypic Hysteresis of *phoQ (T281R)*

**Figure 1:**
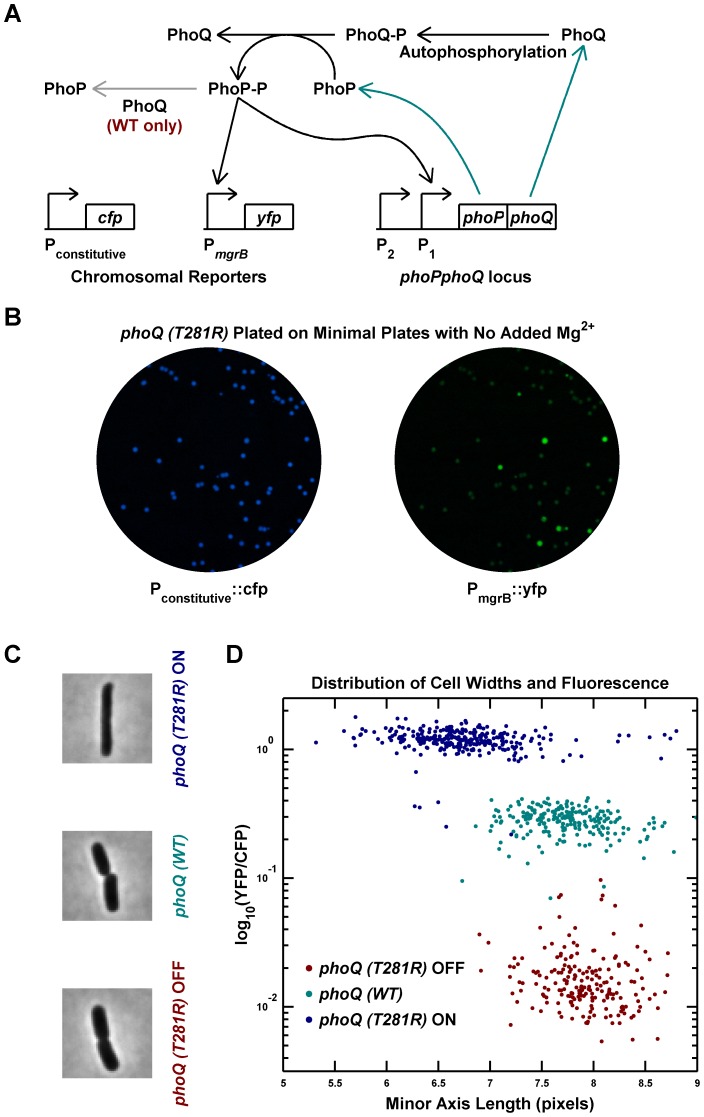
Phenotypic bimodality and hysteresis in the *phoQ (T281R)* mutant. (A) Schematic of the PhoQ/PhoP circuit. The *phoPQ* operon in transcribed and translated to produce PhoP and PhoQ proteins (cyan arrows). Post-tranlational interactions between PhoQ and PhoP generate PhoP-P, which upregulates the expression of the *phoPQ* operon and the *yfp* reporter (solid black arrows). The PhoQ phosphatase activity, which is absent in PhoQ (T281R), is depicted with a gray arrow. (B) Bimodal behavior of YFP colony fluorescence. Panel shows CFP and YFP channel images of a minimal medium plate on which an LB overnight culture of the *phoQ (T281R)* strain was spread. (C and D) Phenotypic hysteresis. Overnight cultures of *phoQ (WT)*, *phoQ (T281R)* OFF, and *phoQ (T281R)* ON strains in LB were diluted 1000-fold into Minimal A medium with 100 µM Mg^2^, grown to mid-exponential phase and imaged by fluorescence microscopy (Methods). The PhoP-P state of the cell was determined by measuring YFP expression driven by the PhoP-P responsive *mgrB* promoter and normalized to CFP expression driven by a constitutive promoter. Cell widths were quantified by fitting phase image masks to ellipses and computing the minor axis length. Representative phase images of the three strains are shown in panel C and reveal morphological differences between OFF and ON cells. Fluorescence and cell width values for each cell are plotted in panel D with indicated colors distinguishing the three strains. Note that *phoQ (T281R)* OFF cells (maroon) have much lower fluorescence and are wider than *phoQ (T281R)* ON cells (blue).

A previous study reported that the *phoQ (T281R)* mutation results in a broad distribution of PhoP-regulated transcription in a population of *E. coli* cells [13]. To explore the origin of this heterogeneity, we engineered a *phoQ (T281R)* strain with the mutation at the native *phoPphoQ* locus. The strain also contained a PhoP-P responsive promoter controlling *yfp* transcription and a constitutive promoter controlling *cfp*, allowing the use of YFP fluorescence to infer PhoP-P levels (Figure 1A and Methods). Two fluorescent colony phenotypes could be discerned on agar plates: YFP-dim, which we designated *phoQ (T281R)* OFF, and YFP-bright, which we designated *phoQ (T281R)* ON (Figure 1B). Considering that each colony is composed of hundreds of millions of cells that originated from a single cell, the appearance of two distinct colony phenotypes suggests that individual cells have two phenotypic states and that these states are heritable.

To determine whether single cells showed a bimodal response, overnight cultures in LB medium were inoculated with OFF and ON colonies, and individual cells were imaged after dilution and growth to mid-exponential phase in minimal medium with 100 µM Mg^2+^ (Figure 1C–D). As with colonies, individual cells could be classified as OFF (low PhoP-P, YFP-dim) or ON (high PhoP-P, YFP-bright) with a ∼60-fold difference in YFP fluorescence between the two states (Figure 1D). The wild-type *phoQ* strain (denoted *phoQ (WT)*) cultured in the same manner showed an intermediate fluorescence, roughly 12-fold higher than the OFF state of the mutant. In addition to the bimodal distribution of phenotypes for the *phoQ (T281R)* strain, we also observed phenotypic hysteresis, i.e., an OFF colony yielded mostly OFF cells, and an ON colony gave rise to mostly ON cells, even though both populations of cells were cultured under the same conditions (Figure 1D). In these experiments, it is likely that the *mgrB* promoter driving *yfp* is near saturation in the ON state and the true change in PhoP-P levels between the OFF and ON states may be higher than 60-fold. Furthermore, since the YFP protein used in this study is stable, the switching of YFP-state from ON to OFF is limited by dilution of YFP due to growth. Consequently, one can see cells with an intermediate YFP-state occasionally even though the PhoP-P levels may actually be low.

Interestingly, the single-cell experiments also revealed a morphological difference between OFF and ON state cells (Figure 1C–D). ON cells have, on average, lower cell-widths (as quantified by the minor axis of the best-fit ellipse) than OFF cells, and the latter are similar to wild-type cells. This is likely an indirect effect of high PhoP-P levels, but we do not know the mechanism.

### Characterization of the OFF state of *phoQ (T281R)*

Why is the phosphatase-deficient mutant not constitutively ON? To gain insights into this question, we formulated a simple mathematical model of the PhoQ/PhoP network that consisted of only three species, viz., PhoP, PhoQ, and PhoP-P and ignored PhoQ-P and intermediate complexes (Text S1). In this model, the kinase rate is a proxy for any factor that can influence the production of PhoP-P from PhoP. Analysis of this model revealed that at high kinase rates, a phosphatase-deficient mutant would indeed be constitutively ON and the network would be monostable (Figure S1A). However, at low kinase rates, the phosphatase-deficient PhoQ/PhoP network could exhibit bistability (exist in OFF and ON states). The bistability results from positive feedback (transcriptional autoregulation, see Figure 1A) and the presence of two non-linearities: (a) the kinase and phosphatase reactions each depend on the product of two concentrations, and (b) the non-linear dependence of *phoPQ* operon transcription on PhoP-P concentration (Text S1). *In vitro* experiments have demonstrated that PhoQ (T281R) is a poorer kinase compared to PhoQ (WT) [13]. The model thus suggests it is both the low kinase activity and the absence of phosphatase activity of the PhoQ (T281R) mutant that facilitates the emergence of bistability. One of the assumptions in our model is that growth-mediated dilution is the dominant mechanism for reduction in concentrations of the stable proteins PhoP, PhoQ and PhoP-P (in the absence of a specific phosphatase). Consequently, growth rate is another parameter that influences the response of the network, with low growth rates leading to slower dilution and a constitutively ON phenotype.

**Figure 2:**
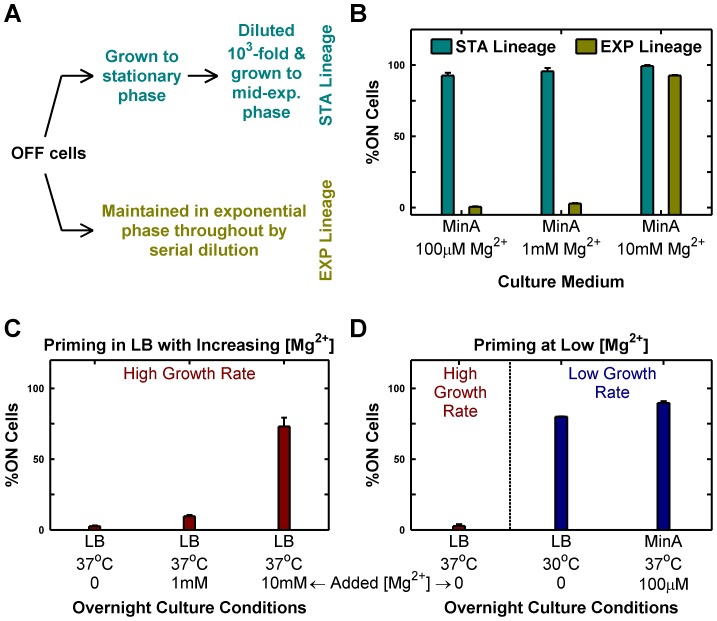
Passage through stationary phase and High Mg^2+^ cause OFF cells to prime to ON state. Priming is defined as the deterministic conversion of OFF cells to the ON state. (A and B) Passage through stationary phase primes OFF cells in minimal medium. Starting from OFF cells, two lineages (STA and EXP) were established in minimal medium with various magnesium concentrations as shown schematically in panel A and in detail in Figure S2. Cells from these lineages were imaged under a microscope as described in Methods and the percentage of ON cells in the mid-exponential culture obtained at the end was plotted (panel B). (C and D) Overnight culture at slow growth rates and high Mg^2+^ results in priming. Overnight cultures inoculated with OFF colonies were set up in indicated conditions, diluted 1000-fold into Minimal A medium with 100 µM Mg^2+^, grown to mid-exponential phase at 37°C (even for 30°C overnight cultures) and imaged under a microscope (Methods). The percentage of ON cells present in the images of mid-exponential cultures is shown. Panel C shows the effect of increasing [Mg^2+^] in LB, while panel D documents the effect of changing growth rate in low [Mg^2+^] media. In panels B, C and D, error bars indicate half the range of two independent experiments. The range is less than 0.5% in the instances where error bars are not visible.

To experimentally validate the insights from the simple model, we reasoned that changing [Mg^2+^] could be used to modulate the PhoP→PhoP-P flux (the kinase rate equivalent). Accordingly, we grew cultures of mostly OFF cells in minimal media with different [Mg^2+^] and maintained them exclusively in the exponential phase by serial dilution (growth rates were similar over the [Mg^2+^] range tested). Surprisingly, we found that high [Mg^2+^] (10 mM) drove all OFF cells to ON, whereas low [Mg^2+^] (100 µM or 1 mM) preserved the OFF state (EXP lineages, Figure 2A–B). This, in effect, represents a reversal of sensitivity to Mg^2+^ in the *phoQ (T281R)* strain as the wild-type strain is repressed (produces lower PhoP-P) by high Mg^2+^. If a portion of the same batch of OFF cells that was maintained in the exponential phase in the EXP lineages was instead passaged through stationary phase (effectively, a slow growth phase) and subsequently diluted and grown to mid-exponential phase, then even the low [Mg^2+^] lineages turned mostly ON (STA lineages, Figure 2A–B). Note that the different outcomes of STA and EXP lineages at low [Mg^2+^] represent yet another manifestation of hysteresis, since both lineages have identical starting points (same culture of OFF cells) and similar end points (mid-exponential cultures with the same [Mg^2+^]), but different history between start and end.

In the present context, the phenotypic hysteresis of *phoQ (T281R)* OFF shown in Figure 1D (i.e. the persistence of these cells in the OFF state) seems anomalous since the OFF cultures were passaged through stationary phase in LB. LB is a rich medium with low (but undetermined) [Mg^2+^]. Consistent with the reversal of Mg^2+^ sensitivity in *phoQ (T281R)*, we find that supplementing LB with high [Mg^2+^] increases conversion of OFF cells to the ON state substantially (Figure 2C). In addition, when we grew LB overnight cultures at 30°C instead of 37°C, we were able to observe OFF→ON conversion at levels comparable to minimal medium with low [Mg^2+^] at 37°C, suggesting that the higher growth rate in LB plays a major role in the absence of OFF→ON conversion at 37°C (Figure 2D). However, we also note that the apparent low OFF→ON conversion in LB at 37°C (Figure 2C) may reflect the competitive advantage of OFF cells over ON ones in LB stationary phase cultures (see below), which would suppress our ability to detect the number of cells that had turned ON.

**Figure 3:**
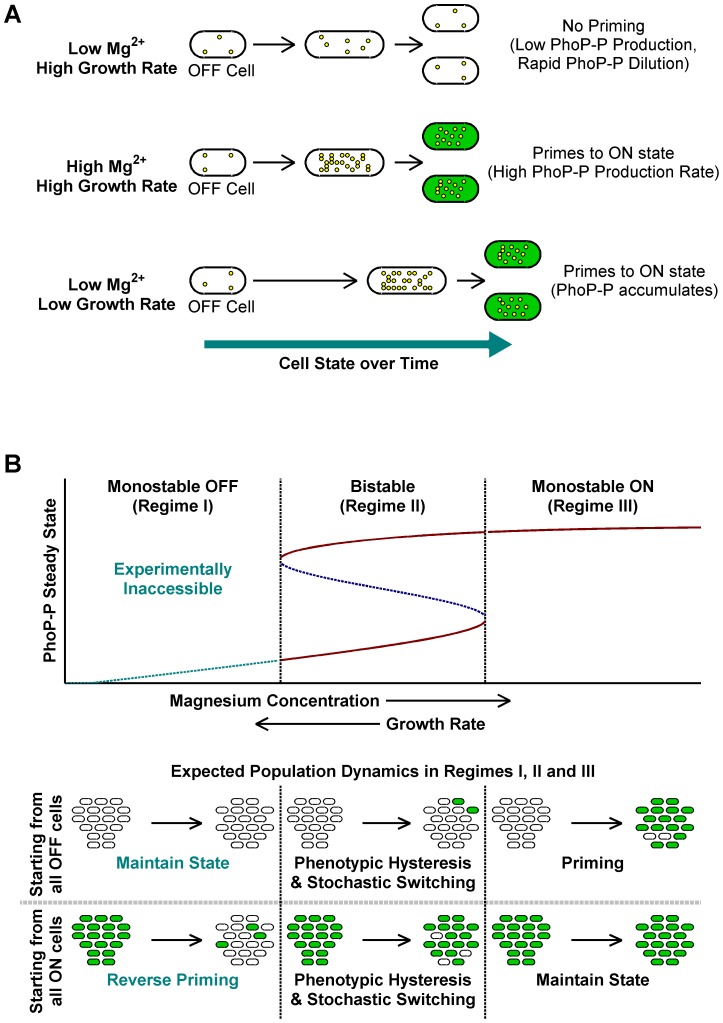
A conceptual framework for priming. (A) Typical fates of OFF cells under different growth conditions. Yellow circles within cells represent PhoP-P molecules. High Mg^2+^ and slow growth rates lead to higher PhoP-P concentrations, which result in conversion of OFF cells to the ON state. (B) Stochastic switching and priming. Typical plot obtained by varying system parameters such as kinase rate, maximal expression rate or growth rate in a mathematical model of the *phoQ (T281R)* network (Text S1) is shown. Steady state PhoP-P values are depicted as a function of the system parameter being varied and the plot can be categorized into three distinct regimes as indicated. Stable OFF and ON state PhoP-P values are plotted in solid maroon. Dashed, blue line represents the unstable intermediate state in the bistable regime. Experimentally inaccessible monostable OFF steady states are shown with a dashed, cyan line. The empirically observed effect of changing magnesium concentrations and growth rate is indicated below the x-axis of the plot. Expected fates of pure OFF and pure ON populations in the three regimes are also illustrated (bottom). The bistable regime is characterized by phenotypic hysteresis and stochastic state switching, whereas priming would be seen in the monostable ON regime.

Taken together, the above results indicate that slow growth histories or high [Mg^2+^] are sufficient for *en masse* conversion of OFF cells to ON (Figure 3A). We call this deterministic conversion from OFF to ON state “priming”.

### Conceptual Framework for the *phoQ (T281R)* Network

To further explore the mechanisms responsible for the phenomena described above, we developed a more detailed mathematical model of the PhoQ/PhoP network consisting of six species (PhoP, PhoP-P, PhoQ, PhoQ-P, and two intermediate protein complexes). While bistability provides an explanation for the observed all-or-none behavior based on deterministic steady-state analysis, it is also possible for a monostable network to exhibit bimodal behavior because of slow kinetics and positive feedback, as has been seen in stochastic models [16], [20], [21]. To compare and contrast these mechanisms, we examined the detailed model using both deterministic steady-state analysis and stochastic simulations (Text S1).

Qualitatively, the steady state behavior of the detailed model is similar to the simpler 3-species model indicating that the simpler model captures the essential features of the network required for bistability. In the model, there are three important kinetic parameters influencing bistability – the kinase rate of PhoQ (T281R), the maximal expression rate of the *phoPQ* operon, and the growth rate of the organism (Text S1). When one of these parameters is varied while keeping all others constant, the system can transition from a monostable OFF regime to a bistable regime and thence to a monostable ON regime (Figure 3B). Note that our model does not incorporate the role of [Mg^2+^] explicitly, but we posit that for PhoQ (T281R), raising [Mg^2+^] leads to an increase in the kinase rate or in the maximal operon expression rate.

Stochastic simulations also largely agree with the deterministic analysis, except that noise-induced bimodality can be observed with parameters in the monostable ON regime close to the bistable regime (Text S1). While noise-induced bimodality and deterministic bistability are different mechanisms, operationally it is difficult to distinguish them without detailed measurement of kinetic parameters *in vivo*. In either case, one would see inheritance of OFF and ON states over several generations.

Within our modeling framework, exponential phase in minimal medium with low [Mg^2+^] can be considered a condition in, or close to, the bistable regime. In this regime, cells retain their state except for rare, stochastic switching events (Figure 3B, Regime II). Slow growth rates or high [Mg^2+^] can independently drive the system towards the monostable ON regime, whereupon OFF cells proceed deterministically towards the ON state (Figure 3B, Regime III). Note that the rate of priming may be kinetically limited, but eventually all cells will turn ON. When these ON cells are sub-cultured in low [Mg^2+^] minimal medium, a bistable (or near-bistable) regime is established again, but hysteresis ensures that cells remain in the ON state as seen in STA lineages in Figure 2B.

### Characteristics of the ON State of *phoQ (T281R)*

Given that a majority of OFF cells can be turned ON by increasing [Mg^2+^] or by passaging through stationary phase, we asked whether there were culture conditions in which an ON population could be deterministically transformed to the OFF state. In other words, we were interested in establishing a monostable OFF regime (Figure 3B, Regime I). According to our model, this could in principle be achieved with low [Mg^2+^] and high growth rates. However, we were unable to observe a monostable OFF regime for exponential growth in minimal medium with 100 µM Mg^2+^ (data not shown) or for growth in LB (Figure 1D). We could not use significantly lower [Mg^2+^] levels without affecting growth rate. These results suggest that it may not be possible to experimentally realize the monostable OFF regime, i.e., the ON state may always be stable. A bistable system with this property is termed irreversible [22]. Note that irreversibility of the system only means that a population of ON cells cannot be deterministically turned OFF. Individual ON cells can still transition to the OFF state in the bistable regime because of stochastic fluctuations (Figure 3B, Regime II). Stochastic switching from the ON to OFF state and its implications are examined later in this study.

**Figure 4:**
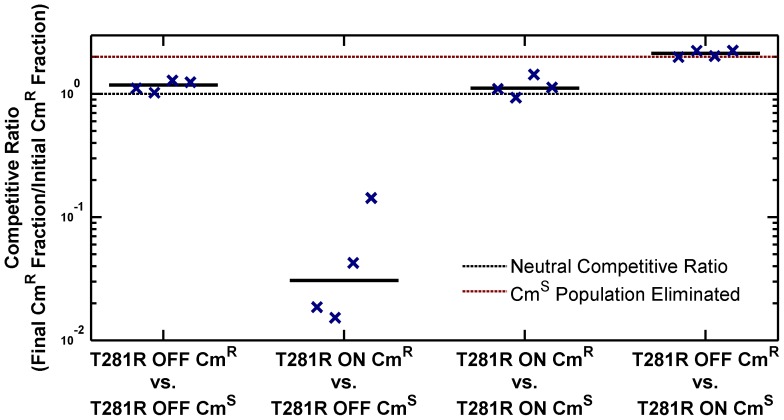
ON cells have a competitive disadvantage in stationary phase in LB. Overnight cultures of chloramphenicol-sensitive (Cm^S^) and chloramphenicol-resistant (Cm^R^) variants of *phoQ (T281R)* OFF and *phoQ (T281R)* ON were set up independently in LB. For each competition experiment, 1 ml of Cm^S^ and Cm^R^ overnight cultures were mixed and co-cultured for an additional 10 hours in stationary phase. Initial and final total and Cm^R^ populations were quantified by plating appropriate dilutions on LB and LB+chloramphenicol plates (see Figure S3 for a detailed protocol). Colony counts were used to compute a competitive ratio (CR), defined as CR = (C(10)L(0))/(C(0)L(10)), where C(T) and L(T) denote the counts on chloramphenicol and LB plates at time T respectively. Note that CR is different from the competitive index (CI) that is frequently used to quantify the outcome of competition experiments (CI is the quotient of final and initial ratios of the population of the two competing strains). In our experimental design, CR has an upper bound of ∼2, whereas CI is, in principle, an unbounded quantity. In competitions between Cm^S^ ON and Cm^R^ OFF cells, the final Cm^S^ population is below the detection limit and CI cannot be computed. Symbols indicate CR values obtained from 4 independent competition experiments. The solid, black line represents the median. Dashed, black line indicates a neutral competitive ratio of 1. Note that a CR of 2 (dashed, maroon line) corresponds to a near-elimination of the Cm^S^ population.

The high PhoP-P level in the ON state has pleiotropic effects on the cell. As presented in Figure 1C–D, cell morphology is affected in the ON state. We also determined that the ON state has a lower fitness in stationary phase in LB. To explore fitness differences between ON and OFF cells, we performed competitions using chloramphenicol resistant (Cm^R^) and sensitive (Cm^S^) *phoQ(T281R)* strains prepared in the ON and OFF states (Figure 4, S3 and Methods). When ON and OFF cells were competed for 10 hours in stationary phase in LB, the ON fraction in the population showed a significant decrease (competitive ratio, Figure 4, columns 2 and 4). In contrast, competitions between Cm^S^ and Cm^R^ OFF cells or between Cm^S^ and Cm^R^ ON cells yielded a near-neutral competitive ratio (Figure 4, columns 1 and 3 respectively).

### Metastability of the ON State and Epigenetic Trapping

To examine stochastic ON→OFF transitions more closely, we performed long-term culture experiments in LB. As mentioned above, a low [Mg^2+^], high growth rate medium such as LB is ideal for observing ON→OFF switching since that growth condition is likely to be close to the theoretical monostable OFF regime. Furthermore, we reasoned that the competitive advantage of OFF cells in stationary phase in LB could be used to amplify the effects of switching and enhance our ability to detect switching events. We established independent lineages by inoculating LB cultures with either *phoQ (T281R)* OFF or *phoQ (T281R)* ON colonies and maintained them through million-fold dilution once per day. The state of the population was assayed by spreading overnight cultures on minimal media plates on alternate days and measuring the fraction of ON (YFP-bright) colonies (Figure S4). This protocol subjected populations to ∼20 generations of exponential growth per day but the majority of time (>14 hours/day) was spent in stationary phase. Furthermore, at least ∼2000 cells were transferred from one day to the next, which meant that even if the fraction of OFF cells in the saturated previous day culture of an ON lineage was as low as 0.1%, there was a ∼90% chance that an OFF cell would be present in the inoculum for the next day culture based on the statistics of binomial sampling.

**Figure 5:**
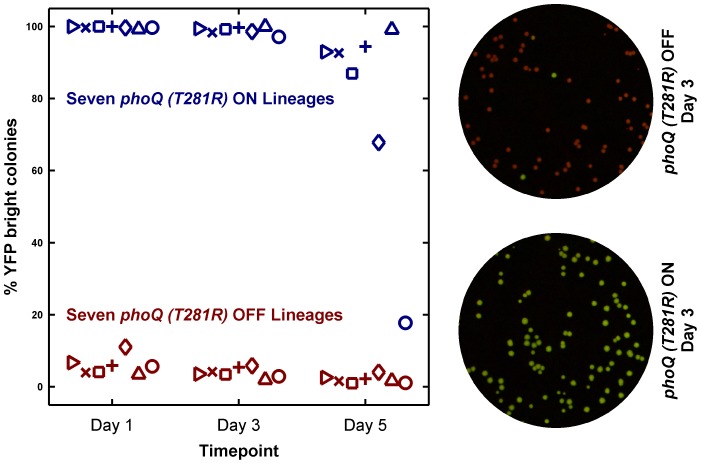
Long-term culturing demonstrates the metastability of the ON state in LB. Seven independent lineages of *phoQ (T281R)* OFF and *phoQ (T281R)* ON were established and maintained as depicted in detail in Figure S4. OFF and ON colonies were inoculated in LB and grown for 24 hours to generate a Day 1 culture. For each lineage, the Day 1 culture was diluted 10^6^-fold to generate a corresponding Day 2 culture, and this procedure was repeated for a total of 5 days. Day 1, 3, and 5 cultures were also diluted and plated in duplicate on minimal medium plates. The mean percentage of YFP-bright colonies on these plates was plotted in maroon (*phoQ (T281R)* OFF) or blue (*phoQ (T281R)* ON) with different symbols representing independent lineages. Representative merged images of Day 3 plates are shown on the right. These were constructed by merging the background-subtracted CFP image with its corresponding background-subtracted YFP image as the red and green channels respectively.

As expected, lineages inoculated with *phoQ (T281R)* OFF yielded mostly OFF colonies on the assay plates (Figure 5). Lineages inoculated with ON colonies, however, showed a different pattern. These remained close to 100% ON for several days (Figure 5, see Day 3 time point), but on Day 5, an appreciable fraction of YFP-dim colonies could be seen in 6 out of 7 lineages. Furthermore, the fraction of YFP-dim colonies obtained in the different lineages was not the same. This divergence highlights both the stochastic nature and the low probability of ON→OFF switching. Since all the ON lineages are likely to converge to a mostly OFF state eventually, the ON lineage in LB is metastable – a long-lived, but not truly stable state due to the competitive advantage of the OFF state. We note that the ON→OFF transitions seen in the ON lineages are unlikely to be the result of mutational events since the YFP-dim colonies obtained in these lineages could be primed ON by overnight growth in minimal medium with 10 mM Mg^2+^ (1 dim colony was tested per lineage, data not shown).

These results show that strong epigenetic inheritance can effectively trap a population in a low-fitness state under some circumstances. Irreversibility enhances this phenomenon by rendering rare, stochastic transitions between states as the fastest means for “escape” from the epigenetic trap.

### *phoQ (T281R)* Properties Influenced by Network Architecture

Having examined the unique characteristics of the *phoQ (T281R)* strain, we asked whether there were any essential features in the PhoQ/PhoP network architecture that enabled a single point mutation to produce such remarkable behavior. Our modeling suggested that bistability (or near-bistability for the stochastic model) in the network was strongly dependent on PhoP-P regulation of both *phoP* and *phoQ* transcription (Text S1). We found that an altered PhoQ/PhoP network where *phoP* transcription is autoregulated but *phoQ (T281R)* transcription is independent of PhoP-P was likely to be monostable, especially if the phosphatase defect in PhoQ (T281R) stemmed from a high dissociation constant of the complex between PhoP-P and PhoQ (an intermediate in the phosphatase reaction) (Text S1 and Figure S1D).

**Figure 6:**
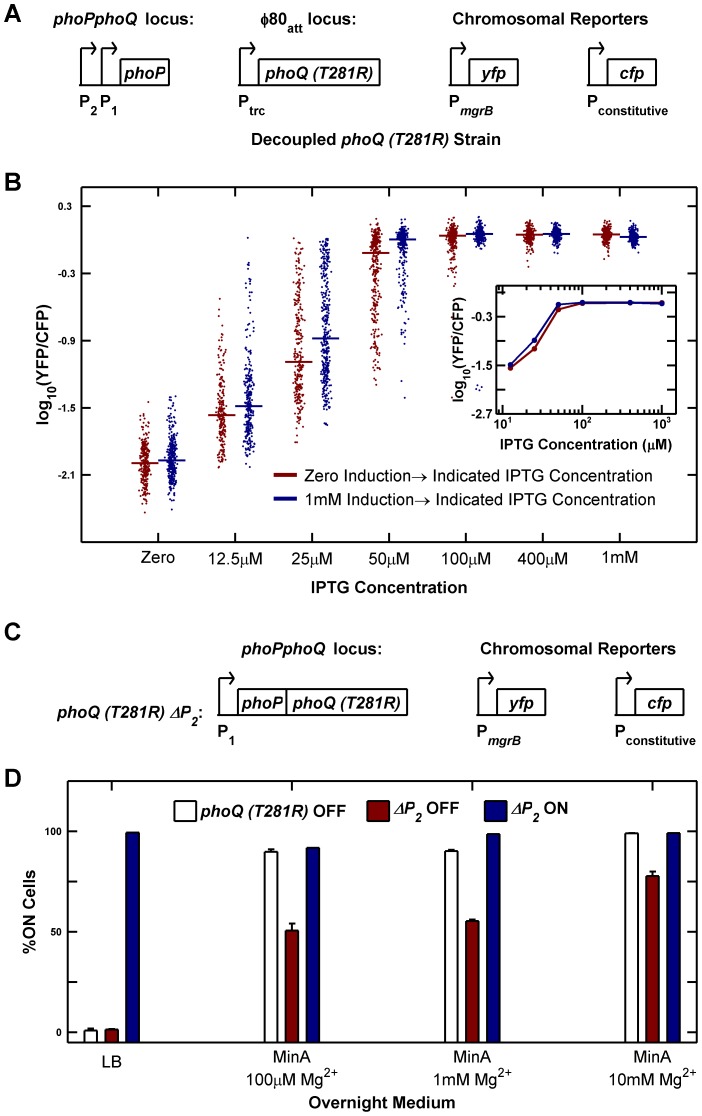
Architectural features of the PhoQ/PhoP network that govern bistability and priming. (A and B) Decoupling of *phoQ (T281R)* expression from PhoP-P control results in loss of bistability. Panel A is a schematic of the decoupled *phoQ (T281R)* strain. *phoP* is driven by its native promoters: the constitutive P_2_ and the PhoP-P responsive P_1_ promoters, whereas expression of *phoQ (T281R)* is governed by the PhoP-P independent, IPTG-inducible P_trc_ promoter. Uninduced and fully induced cultures of the decoupled strain were washed twice in minimal medium without IPTG, diluted 10^5^-fold into Minimal A medium containing 100 µM Mg^2+^ with indicated IPTG concentrations, grown to mid-exponential phase and imaged under a microscope (Methods). YFP/CFP distributions at various induction levels are shown in panel B. For each sample, the median value of YFP/CFP is represented as a horizontal line. Samples derived from uninduced and fully induced cultures are plotted in maroon and blue respectively. Inset shows the median YFP/CFP value as a function of IPTG concentration for samples derived from the induced (blue) and uninduced (maroon) cultures. (C and D) Deletion of the constitutive P_2_ promoter of the *phoPQ* operon reduces priming. Panel C is a schematic of the *ΔP_2_* strain in which the constitutive P_2_ promoter of the *phoPQ* operon had been deleted. Overnight cultures of *phoQ (T281R)* OFF, *ΔP_2_* OFF, and *ΔP_2_* ON strains in indicated medium at 37°C were diluted 1000-fold into Minimal A medium with 100 µM Mg^2+^, grown to mid-exponential phase and imaged under a microscope (Methods). The percentage of ON cells in the images is plotted in panel D. Error bars indicate half the range of two independent experiments. The range is less than 0.5% in the instances where error bars are not visible.

To test whether elimination of PhoP-P dependent transcription of *phoQ (T281R)* abrogated bistability, we constructed a strain in which *phoQ* was deleted from its native locus and *phoQ (T281R)* was inserted at a phage attachment site under the control of the IPTG-inducible (and PhoP-P insensitive) P_trc_ promoter (Figure 6A). We measured YFP/CFP in individual cells after 10 hours of growth (∼15 generations) at various IPTG concentrations starting from uninduced and fully induced populations of cells (Figure 6B). We did not observe any evidence of hysteresis in this strain: cultures started with both fully induced and uninduced cells converge to similar distributions for all IPTG concentrations tested (Figure 6B). Furthermore, distributions spanning intermediate values of YFP/CFP could be observed even after 15 generations of culture (i.e. populations did not converge to distributions with low and high modes). Taken together, these observations suggest that there is no bistability in the decoupled strain. The wide distributions seen at 12.5 µM and 25 µM IPTG can be attributed to a combination of noise in IPTG induction [23], [24] and the sensitivity of the system to induction level in this range (Figure 6B inset) and are also seen in our stochastic simulations (Figure S5D). The slightly lower median of samples derived from the uninduced culture compared with the induced culture could be a result of a stochastic effect that slows down induction kinetics in autoregulated systems [16]. We also determined that a strain similar to the one depicted in Figure 6A, but with *phoQ (T281R)* under the control of its native *phoPQ* promoter instead of P_trc_ exhibited bistability (data not shown).

As documented in Figure 2, one of the defects in the *phoQ (T281R)* strain is that OFF cells transition to the ON state both in response to specific signals such as high [Mg^2+^] and to extraneous factors such as growth rate reduction in stationary phase. We therefore explored whether the *phoQ (T281R)* network could be modified to reduce priming in stationary phase whilst minimally affecting other characteristics of the strain. Based on our model, we predicted that a reduction in the basal level of *phoPQ* transcription (PhoP-P independent transcription) would extend the range of growth rates for which the OFF state was stable, thereby reducing conversion to the ON state at lower growth rates (Text S1 and Figure S1C). To test this prediction, we took advantage of the fact that *phoPphoQ* transcription is driven by two promoters: one is activated by PhoP-P and the other is constitutive [25]. We constructed a variant of the *phoQ (T281R)* strain with the −35 and upstream region of the constitutive P_2_ promoter deleted (Figure 6C). As with the parent strain, we obtained YFP-dim and YFP-bright colonies and designated these *ΔP_2_* OFF and *ΔP_2_* ON respectively. Consistent with the model predictions, only half of *ΔP_2_* OFF cells prime in minimal medium with low [Mg^2+^] compared to ∼90% priming in OFF cells of the parent strain in these conditions (Figure 6D, compare maroon and white bars). The primed fraction in *ΔP_2_* OFF also decreases in minimal medium with high [Mg^2+^], but only to ∼80% indicating that sensitivity to high [Mg^2+^] is mostly retained (Figure 6D, last column). As expected, neither *ΔP_2_* OFF cells nor OFF cells of the parent strain show any priming in LB overnight cultures (Figure 6D, first column). In contrast to the OFF cells, *ΔP_2_* ON cells remain ON in all four culture media indicating that the irreversibility of the system is not affected (Figure 6D, blue bars). We also verified that the deletion of the P_2_ promoter did not adversely affect the PhoP-P responsiveness of the P_1_ promoter by comparing YFP reporters driven by the native *phoPQ* promoter and P_1_ promoter alone and determining that the two reporters behave similarly at high PhoP-P levels (Figure S6).

## Discussion

We have demonstrated that a single point mutation, causing a T281R substitution in the histidine kinase PhoQ, results in a remarkable change in phenotype, with cells exhibiting bistability/bimodality and an inverted response to [Mg^2+^]. Previous modeling work indicates that two-component systems have the potential to exhibit bimodal behavior as a result of stochastic fluctuations in molecular components [16], [20], [21] as well as bistability [26], [27]. Bistability has been observed experimentally in the MprB/MprA two-component system [28], although additional feedback loops not present in simple two-component signaling architectures may be responsible for the bistability in that system [29]. Beyond two-component systems, the response of the *lac* operon to certain gratuitous inducers is another setting where bistability and strong epigenetic inheritance has been observed in bacteria [30], [31]. Like the *lac* operon, we find the OFF and ON states in PhoQ (T281R) can be inherited over many generations. However, we have not been able to find conditions where ON cells can be deterministically converted to an OFF state. Such irreversibility has been seen in the development of *Xenopus* oocytes [32] and is more akin to transient or terminal differentiation seen during bacterial competence or sporulation. Both competence and sporulation are highly regulated phenomena [33] and neither is, strictly speaking, a manifestation of a simple positive-feedback based bistable switch [34], [35]. In this sense, irreversibile bistability is a distinctive feature of the PhoQ (T281R) network among characterized two-component signaling systems.

While bistability and noise-induced bimodality are alternate explanations for the observed behavior of the PhoQ (T281R) network, it may be difficult to differentiate between these mechanisms experimentally. First, in our simulations, we observe noise-induced bimodality only for monostable parameters close to the true bistable regime (Text S1). Thus, distinguishing between these mechanisms would likely require careful measurement of kinetic parameters *in vivo*, which can be challenging even for simple networks. Second, for the mechanism based on noise-induced biomodality, if the timescale for conversion from OFF to ON state in the bimodal regime is tens of generations, then any small fitness difference between the two states can become relevant and even lead to the emergence of bistability, as has been shown in a synthetic circuit [36].

What are the architectural features of the PhoQ/PhoP circuit that enable the T281R mutation to produce such unusual properties? The modeling presented here reveals that the mutant network can readily show bistable behavior when both the kinase and phosphatase activities of the histidine kinase are low (Text S1). A point mutation that simultaneously affects both functions of the histidine kinase can produce a bistable response, as is the case with the T281R mutation, where the kinase activity is low and the phosphatase activity is not detectable. Modeling also indicated that autoregulation of both the response regulator and histidine kinase is required for bistability (Figure 5B). Thus, since *phoP* and *phoQ* are organized as an operon that is driven by a PhoP-P responsive promoter, the architecture of the PhoQ/PhoP network (Figure 1A) is poised to show bistability upon introduction of the T281R mutation in PhoQ. Given that bifunctional histidine kinases and autoregulation are common themes in two-component signaling [6], mutations decreasing phosphatase activity in other histidine kinases may similarly lead to bistable behavior.

Since our model does not explicitly incorporate the role of [Mg^2+^] as an input signal, we can only speculate on the potential mechanism for the inversion of [Mg^2+^]-sensitivity in the mutant. In terms of our model, the inversion can be explained if higher [Mg^2+^] increases the kinase rate of PhoQ (T281R) or increases the maximal expression rate of the *phoPphoQ* operon (potentially through a PhoP-P independent mechanism). Both of these effects could be masked in the wild-type network. For example, an increase in kinase rate at high [Mg^2+^] could be accompanied by a much larger increase in phosphatase rate in PhoQ (WT) resulting in the observed repression of PhoP-P levels under these conditions. Any increase in transcriptional activity is likely to be unnoticed in the wildtype as the output for the wild-type circuit is relatively insensitive to PhoP and PhoQ protein levels for most ranges of magnesium concentration [13]. We should also note that the reversal of input sensitivity may be an idiosyncrasy of the PhoQ/PhoP system and not a general feature of phosphatase mutants of bifunctional histidine kinases.

The *phoQ (T281R)* mutant strain exhibits phenotypic hysteresis, that is, the state of a population depends strongly on the culture history (Figure 1D and 2B). OFF cells retain their state in exponential phase, but prime to the ON state upon passage through stationary phase in minimal medium with low [Mg^2+^] (Figure 2B). In the *lac* operon, slow growth rate has been shown to increase the transition probability from the uninduced to induced state through the accumulation of inducer and the permease protein LacY [37]. A reduction in growth rate of the *phoQ (T281R)* strain can similarly cause PhoP and PhoQ to accumulate because of residual expression of the *phoPphoQ* operon. In addition, PhoP-P would be expected to accumulate from PhoQ (T281R) kinase activity and from the lack of a specific phosphatase. Either mechanism can account for the phenomenon of priming.

Is it possible for strains with wild-type *phoQ* to show bistability? The wild-type PhoQ/PhoP system responds to lowering Mg^2+^ concentrations in a unimodal, graded manner [13]. As long as the loss of PhoP-P due to the phosphatase activity of PhoQ (WT) dominates over the reduction in PhoP-P concentration due to growth-mediated dilution, the network is monostable (Text S1 and ref. [13]). It is conceivable that under highly activating conditions, the phosphatase activity of PhoQ (WT) is sufficiently low, in effect, phenocopying the phosphatase defect in *phoQ (T281R)*. Under these circumstances, our analysis (Text S1) suggests that bistability is possible provided that (1) enough PhoP-P can be produced to begin to saturate the PhoP-P mediated feedback expression of the *phoPQ* operon and (2) the constitutive expression of the operon is sufficiently low (Figure S1B). However, at a Mg^2+^ concentration of 100 µM, which is an activating stimulus for PhoQ (WT), condition (1) is not satisfied in the wild-type network [38]. It is, therefore, possible that the wild-type circuit has evolved to always maintain a significant level of phosphatase activity to avoid bistability.

The phenotypic heterogeneity associated with bistability can improve fitness in fluctuating or unpredictable environments [39], [40]. Bistability also provides a mechanism for storing information about past environmental conditions, and the persistence of this information can be influenced by the network architecture [41]. In the case of *phoQ (T281R)*, however, bistability comes at a fitness cost because of its irreversible nature. Long-term culture experiments in LB showed that a population of ON cells could be trapped in this low-fitness state. In principle, epigenetic trapping can also occur in a reversible system, but in that case there would be environmental conditions in which the population can deterministically transition to the high-fitness state. In an irreversible system, rare stochastic transitions followed by enrichment due to selection are the only means of escaping from the trap.

Interactions between multiple levels of organization in biological systems can result in unexpected or counter-intuitive phenomena. For example, noise at the molecular level can produce bimodal outcomes in a genetic network that is monostable when analyzed as a deterministic system [42]. Likewise, bistability can emerge in a monostable network because of the influence of the output of the network on the growth rate of the organism [36]. In the case of *phoQ (T281R)*, we find that a bistable genetic network gives rise to a stable and a metastable mode since one of the stable states of the network is inherited efficiently, but has a fitness disadvantage. The *phoQ (T281R)* mutation also perfectly illustrates a recent suggestion by Kitano that biological networks may be more sensitive to ‘fail-on’ failures where components function in unexpected ways than to ‘fail-off’ failures where components do not function at all or are removed [43]. While a *phoQ* deletion would be unresponsive to Mg^2+^ levels, it would not show bistability or epigenetic trapping. In contrast, a single point mutation in the robust PhoQ/PhoP signaling module results in a phosphatase-deficient PhoQ protein that gives rise to bistability and an ON state with pleiotropic alterations in cell physiology.

## Methods

### Bacterial Strains and Growth Conditions

Strains and plasmids used in this study are listed in Tables S1 and S2 respectively, and details of their construction are included in Text S2. Table S3 lists primers used for strain construction. A figure-wise list of strains used to collect data is included as Table S4. Freshly streaked *E. coli* strains grown on Miller LB Agar (Fisher BioReagents, Pittsburgh, PA) plates at 37°C were inoculated either in Miller LB broth (Difco - BD, Franklin Lakes, NJ) or in Minimal A medium (MinA, [44]) supplemented with 0.2% glucose, 0.1% casamino acids (Difco - BD, Franklin Lakes, NJ) and with 100 µM, 1 mM or 10 mM MgSO_4_ as indicated. The phenotype of the colony (YFP-dim or YFP-bright) was always verified prior to inoculation. Overnight cultures were grown at 37°C with aeration in a roller drum and for ∼22.5 hours unless otherwise indicated. These were typically diluted 1000-fold into the indicated medium and grown until most of the cultures reached an OD_600_ of 0.2–0.3 for microscopy. Some ON state cultures did not reach an OD_600_ of 0.2 within 4.5 hours of dilution and these were concentrated by gentle centrifugation and resuspension in a smaller volume prior to imaging.

IPTG-induction experiments with the decoupled *phoQ (T281R)* strain (Figure 6A–B) were performed by overnight culture of the strain in MinA/100 µM Mg^2+^ for 12 hours at 37°C and diluting the overnight 1000-fold into two MinA/100 µM Mg^2+^ cultures. After 2.2 hours, one of these cultures was fully induced by adding isopropyl β-D-1-thiogalactopyranoside (IPTG) to a final concentration of 1 mM. Both the induced and uninduced cultures were grown for an additional 2 hours and then washed twice in MinA/100 µM Mg^2+^ by gentle centrifugation and resuspension. Resuspended cultures were then diluted 10^5^-fold into MinA/100 µM Mg^2+^ with indicated IPTG concentrations, grown at 37°C for 10 hours and imaged as described below. For comparing the *phoPQ* reporter and *ΔP_2_* reporter strains (Figure S6), overnight cultures of these strains were grown in MinA/1 mM Mg^2+^ or MinA/10 mM Mg^2+^ for ∼22.5 hours at 37°C, diluted 1000-fold into MinA/1 mM Mg^2+^ or MinA/10 mM Mg^2+^ with indicated IPTG concentrations and imaged as described below.

### Single-Cell Microscopy

Mid-exponential cultures were cooled rapidly in ice-water slurry and streptomycin was added to a final concentration of 250 µg/ml to inhibit further protein synthesis. Imaging was performed on a motorized inverted microscope (Olympus IX81) essentially as described previously [13], [45]. CFP and YFP fluorescence were quantified in single-cells using custom macros written for ImageJ [46]. At least 150 cells were imaged per condition. For quantifying cell-widths, phase images were thresholded and used to segment individual cells. The minor axis of the best-fit ellipse for each identified cell was used as a measure of cell-width.

### Fluorescence Imaging of Plates

Overnight cultures were diluted 10^6^-fold in MinA supplemented with 0.2% glucose and 0.1% casamino acids. 100 µl of the diluted culture was spread on 1.5% agar plates composed of MinA with 0.2% glucose, 0.1% casamino acids, and no added MgSO_4_. These plates were incubated at 37°C for 20 hours and imaged with a home-built fluorescence illuminator as described previously [15].

### Competition Experiments

Freshly streaked colonies of indicated chloramphenicol-sensitive (Cm^S^) and chloramphenicol-resistant (Cm^R^) strains were inoculated in LB medium and grown at 37°C for 20 hours with aeration in a roller drum. At the beginning of the competition, these saturated cultures were mixed 1∶1 by volume and combined cultures were grown for another 10 hours at 37°C. Total and Cm^R^ colony forming units (cfus) at the beginning and end of the competition were determined by spreading dilutions on LB Agar plates without antibiotic and with 15 µg/ml chloramphenicol respectively.

### Modeling of the *phoQ (T281R)* Network

The *phoQ (T281R)* network was mathematically modeled as described in Text S1. Symbolic manipulations and numerical computations were performed using custom code written in MATLAB (Mathworks, Natick, MA) or C programming language. The region of bistability seen in deterministic models is shown in Figures S7, S8, S9, and representative results from stochastic simulations are shown in Figure S5. Parameters and reactions used in stochastic simulations are presented in Tables S5 and S6.

## Supporting Information

Figure S1 Modeling of the PhoQ/PhoP network reveals various bifurcations responsible for experimentally observed system behaviors. (A) Bifurcation responsible for emergence of OFF and ON states. A simple three-species model of the PhoQ/PhoP network (Text S1) was used to examine the effects of varying phosphatase rates at a given kinase rate. Steady state P* values at high (left) and low kinase rates (right) were plotted as a function of the phosphatase rate. At low kinase rates, there is a bifurcation which gives rise to OFF and ON stable states and an intermediate unstable state. Filled circles depict potential steady state scenarios in *phoQ (WT)* (high phosphatase, left panel) and *phoQ (T281R)* (low phosphatase, right panel). Dashed, horizontal, black line indicates the wild-type steady state. (B) Detailed modeling of the *phoQ (T281R)* mutant shows that bistability can be achieved at sufficiently low constitutive *phoPQ* transcription. A detailed model of the PhoQ/PhoP network (Text S1) was analyzed for steady state behavior. Steady state P* values were plotted as a function of constitutive *phoPQ* transcription (V_0_). (C) Reduction of V_0_ extends the bistable regime to lower growth rates. In the detailed model, reduction in growth rate is equivalent to upscaling of kinetic parameters (Text S1). The bifurcation diagram as a function of growth rate was plotted for high (top) and low (below) V_0_. In either case, at sufficiently low growth rates, the ON state is the only stable state. (D) Modified PhoQ/PhoP network with autoregulated *phoP* and inducible *phoQ (T281R)* shows bistability only when the dissociation constant of the [P*Q] complex is low. Steady state P* values were computed for the modified PhoQ/PhoP network (Text S1) at high and low dissociation constants for the [P*Q] complex and were plotted as a function of total PhoQ (Q_tot_). Bistability is seen for Q_tot_ values between the black, vertical, dashed lines. In all panels, stable and unstable steady states are plotted in solid maroon and dashed blue respectively. Steady state values less than 10^−4^ are plotted as 10^−4^.
(PDF)

Figure S2 Protocol for generation of STA and EXP lineages. Details of the protocol used to generate STA and EXP lineages in Figures 2A and 2B are depicted. An OFF-state population was prepared by inoculating a colony of *phoQ (T281R)* OFF in LB and growing overnight. The overnight culture was diluted 1000-fold into minimal media with either 100 µM, 1 mM or 10 mM Mg^2+^. The resulting cultures were grown to mid-exponential phase and used to establish two lineages: the EXP lineage, which was maintained throughout in exponential phase by serial dilutions, and the STA lineage in which the mid-exponential culture was allowed to saturate and the saturated culture was diluted 1000-fold and grown to mid-exponential phase.
(PDF)

Figure S3 Protocol for competition experiments in stationary phase in LB medium. Details of the protocol used to perform competition experiments in Figure 4 are depicted. Overnight cultures of chloramphenicol-sensitive (Cm^S^) and chloramphenicol-resistant (Cm^R^) variants of *phoQ (T281R)* OFF and *phoQ (T281R)* ON were set up independently in LB and grown at 37°C for 20 hours. For each competition experiment, 1 ml of Cm^S^ and Cm^R^ overnight cultures were mixed and the mixed population was grown at 37°C for an additional 10 hours. Initial and final ratios of total and Cm^R^ populations were determined by plating on LB and LB+15 µg/ml chloramphenicol plates.
(PDF)

Figure S4 Protocol for demonstrating metastability of the ON state in LB. Details of the long-term culture experiment in Figure 5 are depicted. An LB culture inoculated with *phoQ (T281R)* OFF or *phoQ(T281R)* ON was grown at 37°C for 24 hours (Day 1 culture). The Day 1 culture was diluted 10^6^-fold to generate the corresponding Day 2 culture. This procedure was repeated for a total of 5 days. To determine the population state, cultures from Days 1, 3 and 5 were also diluted and plated in duplicate on minimal plates with no added Mg^2+^. These plates were imaged after 20 hours incubation at 37°C (Methods).
(PDF)

Figure S5 Stochastic simulations of *phoQ (T281R)* mutant. Stochastic simulations were performed on a detailed model of *phoQ (T281R)* mutant using Gillespie's algorithm (Text S1). (A) The stochastic model was analyzed in the deterministic limit to obtain the steady state number of PhoP-P molecules. These are plotted as a function of the maximal feedback transcription rate (C_P1_). Stable OFF and ON states are plotted in maroon and olive green respectively. Dashed blue lines represent unstable steady states. Four representative C_P1_ values were identified for stochastic simulations and are indicated with vertical, dashed, black lines. (B) Stochastic simulations using parameter sets corresponding to the four values indicated in panel (A). For each parameter set, simulations were performed starting from an OFF state (maroon) or ON state (olive green) and the final PhoP-P number in independent runs was plotted. Runs in which stochastic switching from the ON to OFF state was observed in the bistable regime are depicted as crosses. The wide distribution seen in parameter set 3 starting from the OFF state is characteristic of noise-induced bimodality. (C) Priming in the stochastic model. The effect of growth rate and P_2_ promoter deletion on priming were examined using stochastic simulations. Simulations were performed starting from an OFF state with different combinations of growth rate and the promoter driving *phoPQ* operon (column 1 is identical to parameter set 3 in panels A and B) and the average number of PhoP-P molecules in the last generation of the simulation was plotted. P_2_P_1_ is the native *phoPQ* promoter, whereas P_1_ denotes the ΔP_2_ construct (Figure 6C). The horizontal, black line represents the threshold above which cells are considered primed. The percentage of runs in which priming is observed is indicated. (D) Stochastic simulation of the decoupled *phoQ (T281R)* strain. Stochastic simulations of the decoupled strain (Figure 6A) were performed at various values of *phoQ* transcription rate (i.e., at different levels of induction) starting from an uninduced (maroon) or a fully induced (olive green) state and the final number of PhoP-P molecules was plotted. In panels B–D, at least 1000 independent simulation runs were performed per unique parameter/initial condition combination. Horizontal maroon or olive green lines represent the average value across all independent runs for that condition. Simulations were run for 35 generations in panels B–C, and for 15 generations in panel D.
(PDF)

Figure S6 Deletion of constitutive promoter of the *phoPQ* operon does not affect feedback regulation through PhoP-P. (A) Schematic of the two strains used to determine the effect of deletion of the constitutive P_2_ promoter on PhoP-P feedback. Both strains have the *phoP*-*phoQ (T281R)* operon driven by the IPTG-inducible P_trc_ promoter and identical constitutive *cfp* reporters. The strains differ in the PhoP-P responsive *yfp* reporter. The *phoPQ* Reporter strain (top) has *yfp* driven by an intact *phoPQ* promoter. The *ΔP_2_* Reporter strain (bottom) has *yfp* driven by the PhoP-P responsive P_1_ promoter alone. The portion of the *phoPQ* promoter left intact in the *ΔP_2_* Reporter strain is identical to the *ΔP_2_* strain depicted in Figure 6C. PhoP-P levels in both reporter strains can be varied by inducing the *phoPQ* operon with IPTG. (B) Behavior of *phoPQ* and *ΔP_2_* reporters as a function of PhoP-P levels. Overnight cultures of the *phoPQ* and *ΔP_2_* Reporter strains in Minimal A medium with 1 mM Mg^2+^ (left) or 10 mM Mg^2+^ (right) were diluted 1000-fold into Minimal A medium with 1 mM Mg^2+^ or 10 mM Mg^2+^ with different IPTG concentrations (filled circles). These cultures were grown to mid-exponential phase (4.0 hours of growth) and then YFP and CFP images were taken under the microscope (Methods). The geometric mean of the YFP/CFP distribution was plotted as a function of the IPTG concentration of the culture for both the *phoPQ* Reporter Strain (maroon) and the *ΔP_2_* Reporter strain (blue). At high levels of IPTG induction, which correspond to high PhoP-P levels, the two PhoP-P reporters behave similarly suggesting that PhoP-P binding is largely unaffected by the deletion of the constitutive promoter. Note that there is no bistable behavior in either strain since there is no autoregulation.
(PDF)

Figure S7 Region of bistability in a simple model of the PhoQ/PhoP network. Steady state behavior of a simple model of the PhoQ/PhoP network was analyzed (Text S1) to determine parameter values for which the network showed bistability. The bistable regime is plotted in black. In the monostable regime, the steady state value of P* is plotted according to the color scale on the top. Steady state values less than 10^−4^ are plotted as 10^−4^. The figure is divided into a 4×4 grid of panels. In each panel, the phosphatase rate (k_p_) is varied along the x-axis, whereas feedback strength (V_f_) is varied along the y-axis. λ = 0.02 for all panels. The remaining parameter values are indicated on the top of each panel. See Text S1 for definitions of parameters.
(PDF)

Figure S8 Region of bistability in a detailed model of the *phoQ (T281R)* mutant. Steady state behavior of a detailed model of the *phoQ (T281R)* network was analyzed (Text S1) to determine parameter values for which the network showed bistability. The bistable regime is plotted in black. In the monostable regime, the steady state value of P* is plotted according to the color scale on the top. Steady state values less than 10^−4^ are plotted as 10^−4^. The figure is divided into a 8×8 grid of panels. In each panel, the constitutive promoter strength (V_0_) is varied along the x-axis, whereas feedback strength (V_f_) is varied along the y-axis. λ = 0.02 for all panels. Parameters common to each pair of rows (or columns) are indicated on the left (or top). The remaining parameter values are indicated on the top of each panel. See Text S1 for definitions of parameters.
(PDF)

Figure S9 Region of bistability in a model where *phoQ (T281R)* expression is decoupled from PhoP-P control. Steady state behavior of a modified PhoQ/PhoP network with autoregulated *phoP* and constitutive *phoQ (T281R)* was analyzed (Text S1) to determine parameter values for which the network showed bistability. The bistable regime is plotted in black. In the monostable regime, the steady state value of P* is plotted according to the color scale on the top. Steady state values less than 10^−4^ are plotted as 10^−4^. The figure is divided into an 8×8 grid of panels. In each panel, the total concentration of PhoQ (Q_tot_) is varied along the x-axis, whereas the constitutive promoter strength (V_0_) is varied along the y-axis. λ = 0.02 and k_cons_ = 1 for all panels. Parameters common to each pair of rows (or columns) are indicated on the left (or top). The remaining parameter values are indicated on the top of each panel. See Text S1 for definitions of parameters.
(PDF)

Table S1 List of strains.
(PDF)

Table S2 List of plasmids.
(PDF)

Table S3 List of primers.
(PDF)

Table S4 Figure-wise list of strains.
(PDF)

Table S5 Parameters and reactions for simulation of the autoregulated stochastic model.
(PDF)

Table S6 Parameters and reactions for simulation of the decoupled stochastic model.
(PDF)

Text S1 Modeling the PhoQ/PhoP network.
(PDF)

Text S2 Details of strains and strain constructions.
(PDF)
