# Supplementary figures and images for: The Architecture of a Prototypical Bacterial Signaling Circuit Enables a Single Point Mutation to Confer Novel Network Properties
Source: PLoS Genet. 2013 Aug 22;9(8):e1003706. doi: 10.1371/journal.pgen.1003706 (PMC3750022; doi:10.1371/journal.pgen.1003706)

Figure S1

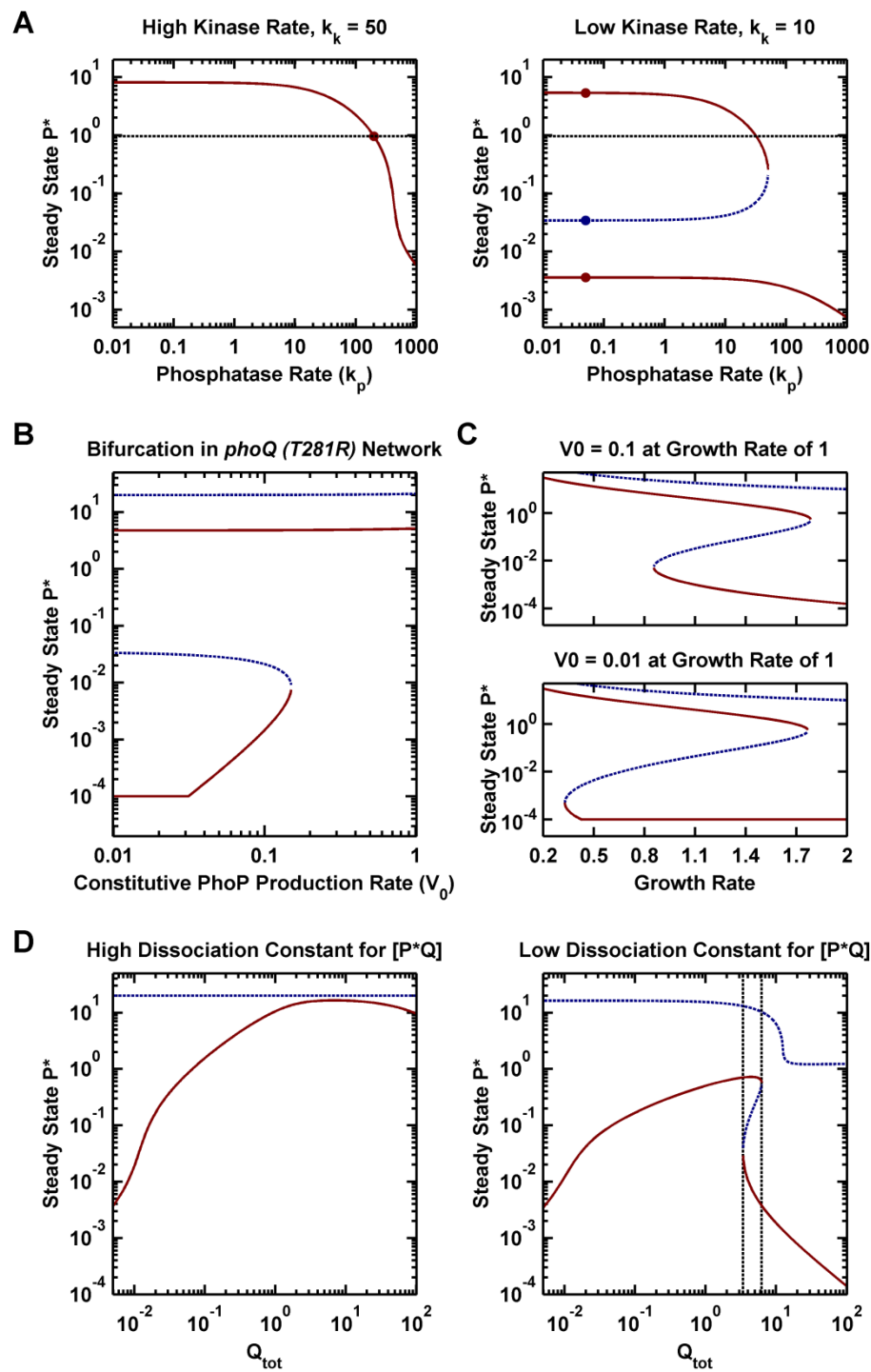

Supplement: Figure S1 — Modeling of the PhoQ/PhoP network reveals various bifurcations responsible for experimentally observed system behaviors. (A) Bifurcation responsible for emergence of OFF and ON states. A simple three-species model of the PhoQ/PhoP network (Text S1) was used to examine the effects of varying phosphatase rates at a given kinase rate. Steady state P* values at high (left) and low kinase rates (right) were plotted as a function of the phosphatase rate. At low kinase rates, there is a bifurcation which gives rise to OFF and ON stable states and an intermediate unstable state. Filled circles depict potential steady state scenarios in phoQ (WT) (high phosphatase, left panel) and phoQ (T281R) (low phosphatase, right panel). Dashed, horizontal, black line indicates the wild-type steady state. (B) Detailed modeling of the phoQ (T281R) mutant shows that bistability can be achieved at sufficiently low constitutive phoPQ transcription. A detailed model of the PhoQ/PhoP network (Text S1) was analyzed for steady state behavior. Steady state P* values were plotted as a function of constitutive phoPQ transcription (V0). (C) Reduction of V0 extends the bistable regime to lower growth rates. In the detailed model, reduction in growth rate is equivalent to upscaling of kinetic parameters (Text S1). The bifurcation diagram as a function of growth rate was plotted for high (top) and low (below) V0. In either case, at sufficiently low growth rates, the ON state is the only stable state. (D) Modified PhoQ/PhoP network with autoregulated phoP and inducible phoQ (T281R) shows bistability only when the dissociation constant of the [P*Q] complex is low. Steady state P* values were computed for the modified PhoQ/PhoP network (Text S1) at high and low dissociation constants for the [P*Q] complex and were plotted as a function of total PhoQ (Qtot). Bistability is seen for Qtot values between the black, vertical, dashed lines. In all panels, stable and unstable steady states are plotted in solid m [file pgen.1003706.s001.pdf]

Figure S2

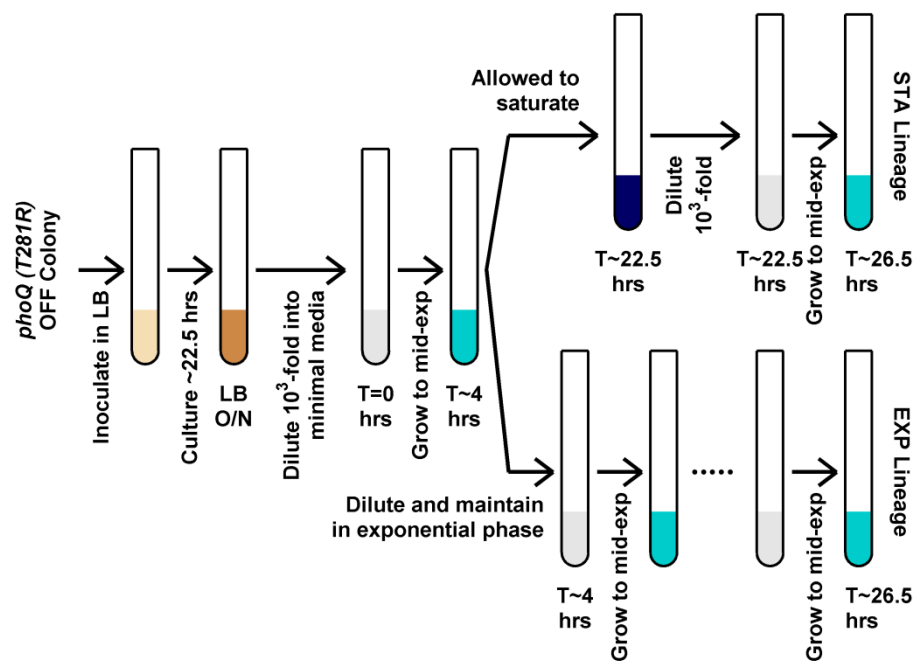

Supplement: Figure S2 — Protocol for generation of STA and EXP lineages. Details of the protocol used to generate STA and EXP lineages in Figures 2A and 2B are depicted. An OFF-state population was prepared by inoculating a colony of phoQ (T281R) OFF in LB and growing overnight. The overnight culture was diluted 1000-fold into minimal media with either 100 µM, 1 mM or 10 mM Mg2+. The resulting cultures were grown to mid-exponential phase and used to establish two lineages: the EXP lineage, which was maintained throughout in exponential phase by serial dilutions, and the STA lineage in which the mid-exponential culture was allowed to saturate and the saturated culture was diluted 1000-fold and grown to mid-exponential phase. (PDF) [file pgen.1003706.s002.pdf]

Figure S3

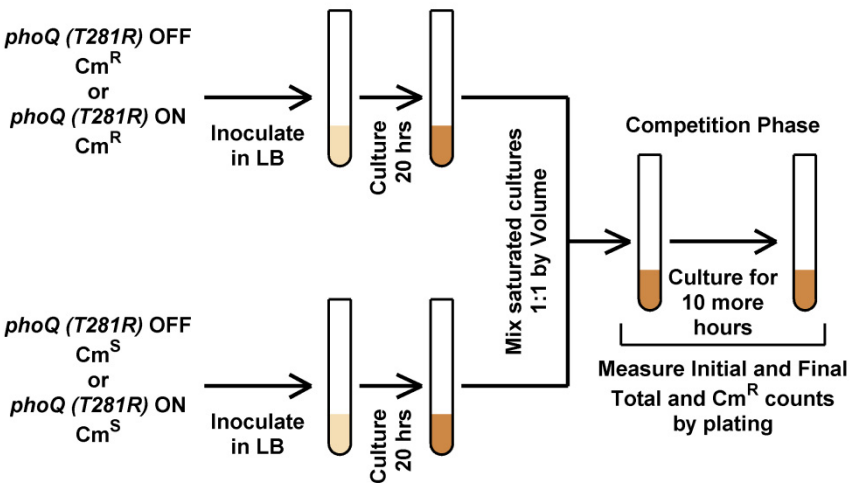

Supplement: Figure S3 — Protocol for competition experiments in stationary phase in LB medium. Details of the protocol used to perform competition experiments in Figure 4 are depicted. Overnight cultures of chloramphenicol-sensitive (CmS) and chloramphenicol-resistant (CmR) variants of phoQ (T281R) OFF and phoQ (T281R) ON were set up independently in LB and grown at 37°C for 20 hours. For each competition experiment, 1 ml of CmS and CmR overnight cultures were mixed and the mixed population was grown at 37°C for an additional 10 hours. Initial and final ratios of total and CmR populations were determined by plating on LB and LB+15 µg/ml chloramphenicol plates. (PDF) [file pgen.1003706.s003.pdf]

Figure S4

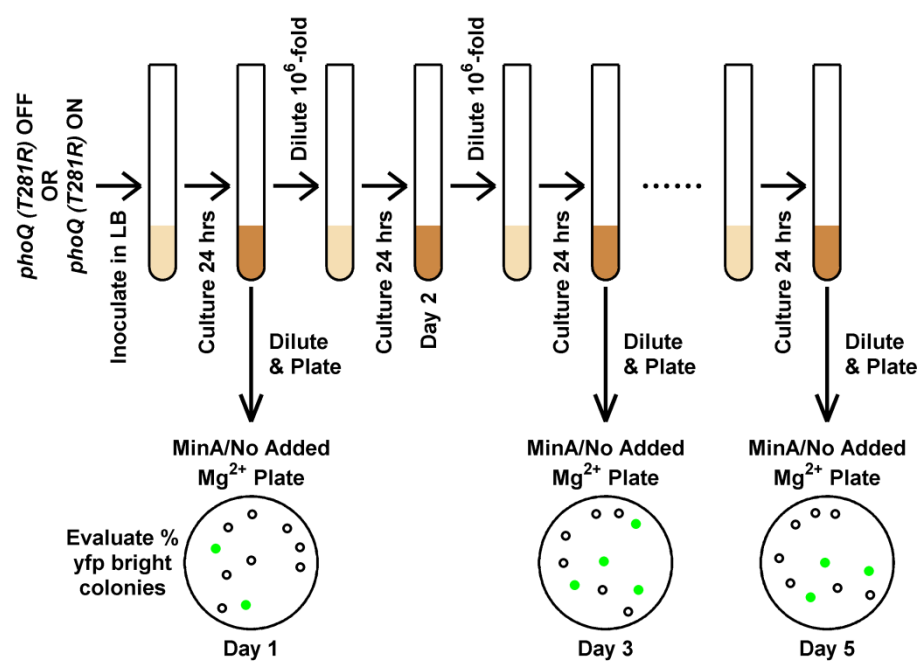

Supplement: Figure S4 — Protocol for demonstrating metastability of the ON state in LB. Details of the long-term culture experiment in Figure 5 are depicted. An LB culture inoculated with phoQ (T281R) OFF or phoQ(T281R) ON was grown at 37°C for 24 hours (Day 1 culture). The Day 1 culture was diluted 106-fold to generate the corresponding Day 2 culture. This procedure was repeated for a total of 5 days. To determine the population state, cultures from Days 1, 3 and 5 were also diluted and plated in duplicate on minimal plates with no added Mg2+. These plates were imaged after 20 hours incubation at 37°C (Methods). (PDF) [file pgen.1003706.s004.pdf]

Figure S5

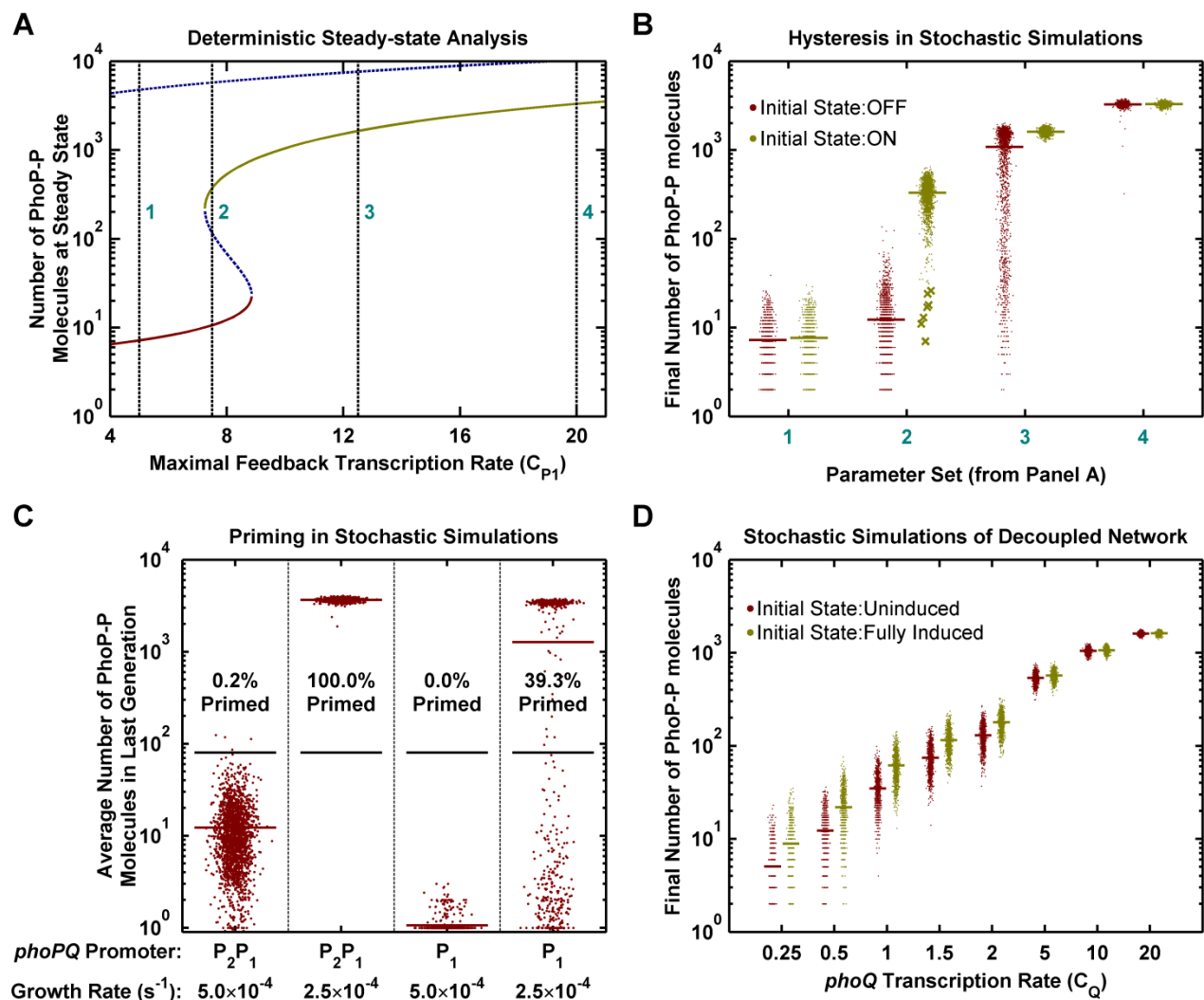

Supplement: Figure S5 — Stochastic simulations of phoQ (T281R) mutant. Stochastic simulations were performed on a detailed model of phoQ (T281R) mutant using Gillespie's algorithm (Text S1). (A) The stochastic model was analyzed in the deterministic limit to obtain the steady state number of PhoP-P molecules. These are plotted as a function of the maximal feedback transcription rate (CP1). Stable OFF and ON states are plotted in maroon and olive green respectively. Dashed blue lines represent unstable steady states. Four representative CP1 values were identified for stochastic simulations and are indicated with vertical, dashed, black lines. (B) Stochastic simulations using parameter sets corresponding to the four values indicated in panel (A). For each parameter set, simulations were performed starting from an OFF state (maroon) or ON state (olive green) and the final PhoP-P number in independent runs was plotted. Runs in which stochastic switching from the ON to OFF state was observed in the bistable regime are depicted as crosses. The wide distribution seen in parameter set 3 starting from the OFF state is characteristic of noise-induced bimodality. (C) Priming in the stochastic model. The effect of growth rate and P2 promoter deletion on priming were examined using stochastic simulations. Simulations were performed starting from an OFF state with different combinations of growth rate and the promoter driving phoPQ operon (column 1 is identical to parameter set 3 in panels A and B) and the average number of PhoP-P molecules in the last generation of the simulation was plotted. P2P1 is the native phoPQ promoter, whereas P1 denotes the ΔP2 construct (Figure 6C). The horizontal, black line represents the threshold above which cells are considered primed. The percentage of runs in which priming is observed is indicated. (D) Stochastic simulation of the decoupled phoQ (T281R) strain. Stochastic simulations of the decoupled strain (Figure 6A) were performed at various values of phoQ transcrip [file pgen.1003706.s005.pdf]

Figure S6

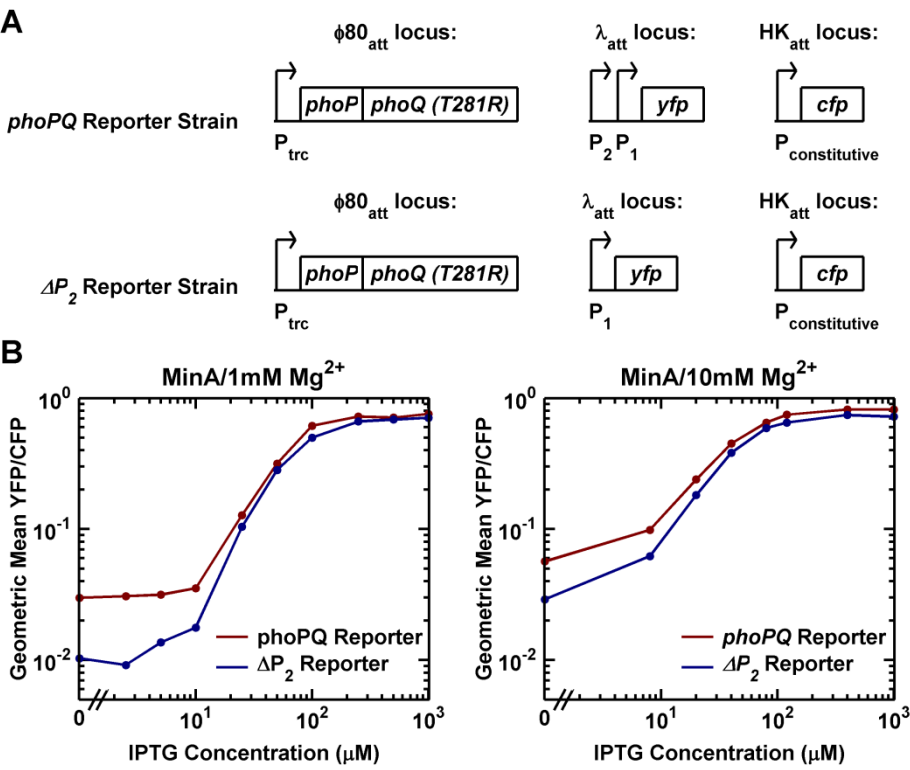

Supplement: Figure S6 — Deletion of constitutive promoter of the phoPQ operon does not affect feedback regulation through PhoP-P. (A) Schematic of the two strains used to determine the effect of deletion of the constitutive P2 promoter on PhoP-P feedback. Both strains have the phoP-phoQ (T281R) operon driven by the IPTG-inducible Ptrc promoter and identical constitutive cfp reporters. The strains differ in the PhoP-P responsive yfp reporter. The phoPQ Reporter strain (top) has yfp driven by an intact phoPQ promoter. The ΔP2 Reporter strain (bottom) has yfp driven by the PhoP-P responsive P1 promoter alone. The portion of the phoPQ promoter left intact in the ΔP2 Reporter strain is identical to the ΔP2 strain depicted in Figure 6C. PhoP-P levels in both reporter strains can be varied by inducing the phoPQ operon with IPTG. (B) Behavior of phoPQ and ΔP2 reporters as a function of PhoP-P levels. Overnight cultures of the phoPQ and ΔP2 Reporter strains in Minimal A medium with 1 mM Mg2+ (left) or 10 mM Mg2+ (right) were diluted 1000-fold into Minimal A medium with 1 mM Mg2+ or 10 mM Mg2+ with different IPTG concentrations (filled circles). These cultures were grown to mid-exponential phase (4.0 hours of growth) and then YFP and CFP images were taken under the microscope (Methods). The geometric mean of the YFP/CFP distribution was plotted as a function of the IPTG concentration of the culture for both the phoPQ Reporter Strain (maroon) and the ΔP2 Reporter strain (blue). At high levels of IPTG induction, which correspond to high PhoP-P levels, the two PhoP-P reporters behave similarly suggesting that PhoP-P binding is largely unaffected by the deletion of the constitutive promoter. Note that there is no bistable behavior in either strain since there is no autoregulation. (PDF) [file pgen.1003706.s006.pdf]

Figure S7

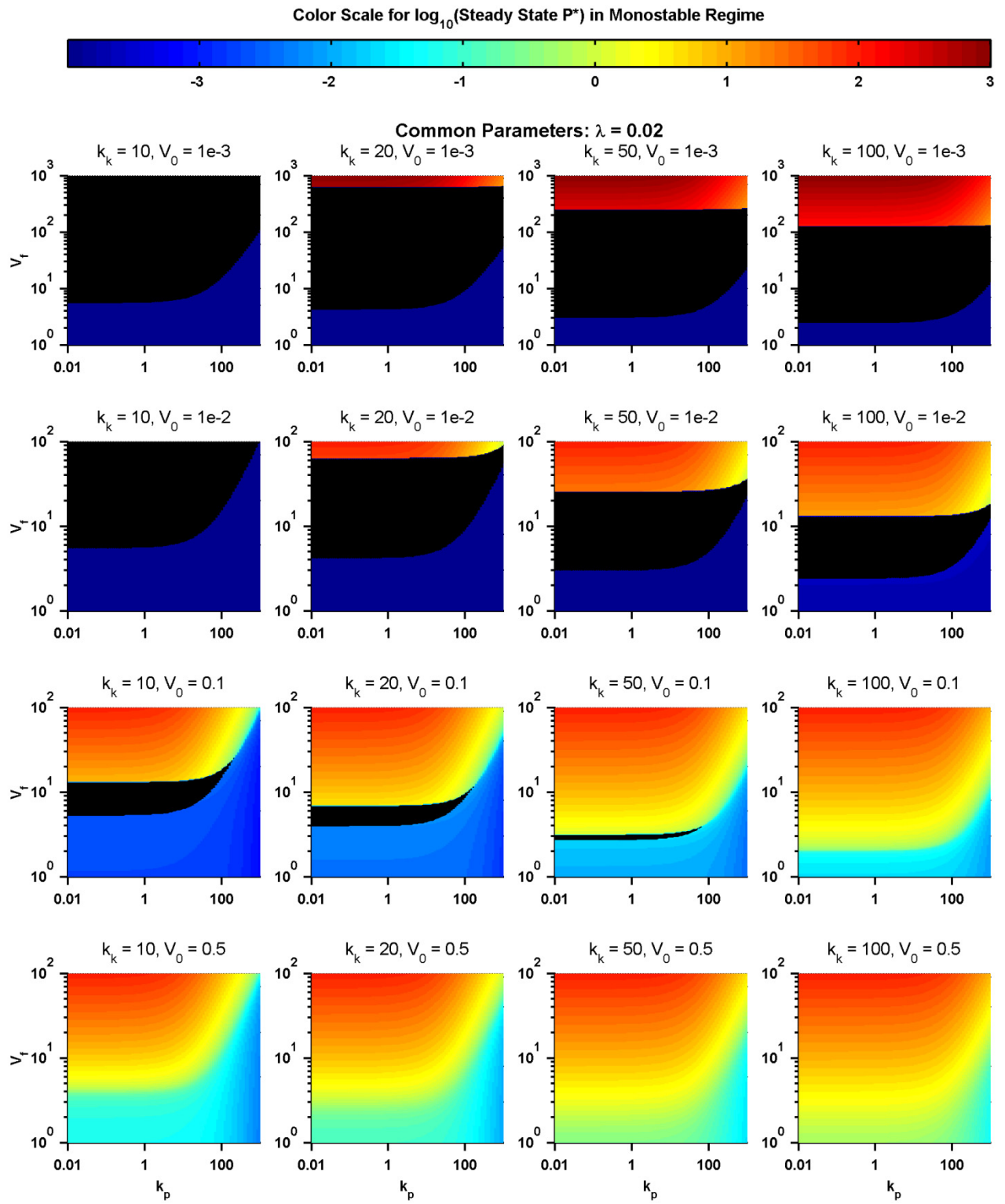

Supplement: Figure S7 — Region of bistability in a simple model of the PhoQ/PhoP network. Steady state behavior of a simple model of the PhoQ/PhoP network was analyzed (Text S1) to determine parameter values for which the network showed bistability. The bistable regime is plotted in black. In the monostable regime, the steady state value of P* is plotted according to the color scale on the top. Steady state values less than 10−4 are plotted as 10−4. The figure is divided into a 4×4 grid of panels. In each panel, the phosphatase rate (kp) is varied along the x-axis, whereas feedback strength (Vf) is varied along the y-axis. λ = 0.02 for all panels. The remaining parameter values are indicated on the top of each panel. See Text S1 for definitions of parameters. (PDF) [file pgen.1003706.s007.pdf]

Figure S8

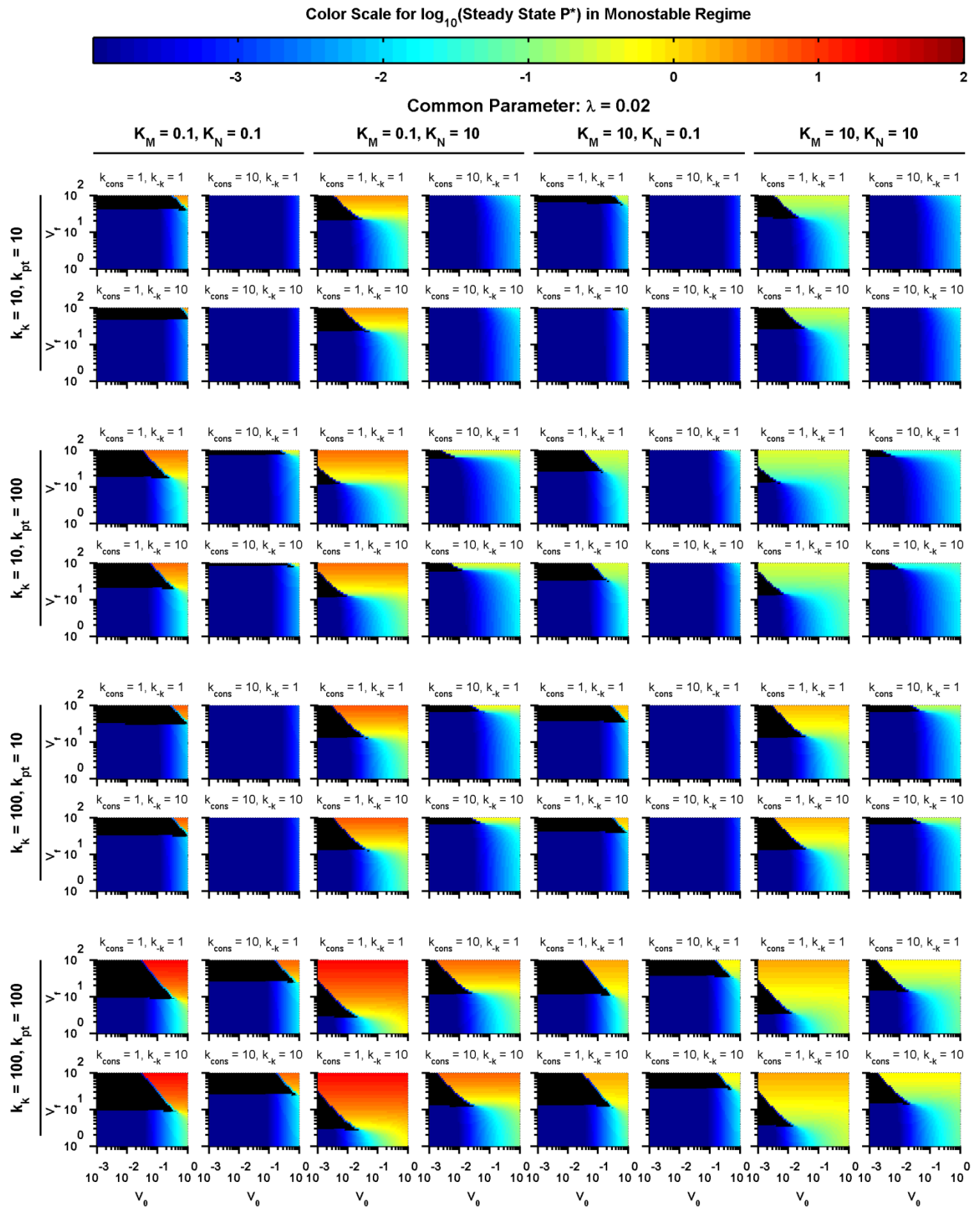

Supplement: Figure S8 — Region of bistability in a detailed model of the phoQ (T281R) mutant. Steady state behavior of a detailed model of the phoQ (T281R) network was analyzed (Text S1) to determine parameter values for which the network showed bistability. The bistable regime is plotted in black. In the monostable regime, the steady state value of P* is plotted according to the color scale on the top. Steady state values less than 10−4 are plotted as 10−4. The figure is divided into a 8×8 grid of panels. In each panel, the constitutive promoter strength (V0) is varied along the x-axis, whereas feedback strength (Vf) is varied along the y-axis. λ = 0.02 for all panels. Parameters common to each pair of rows (or columns) are indicated on the left (or top). The remaining parameter values are indicated on the top of each panel. See Text S1 for definitions of parameters. (PDF) [file pgen.1003706.s008.pdf]

Figure S9

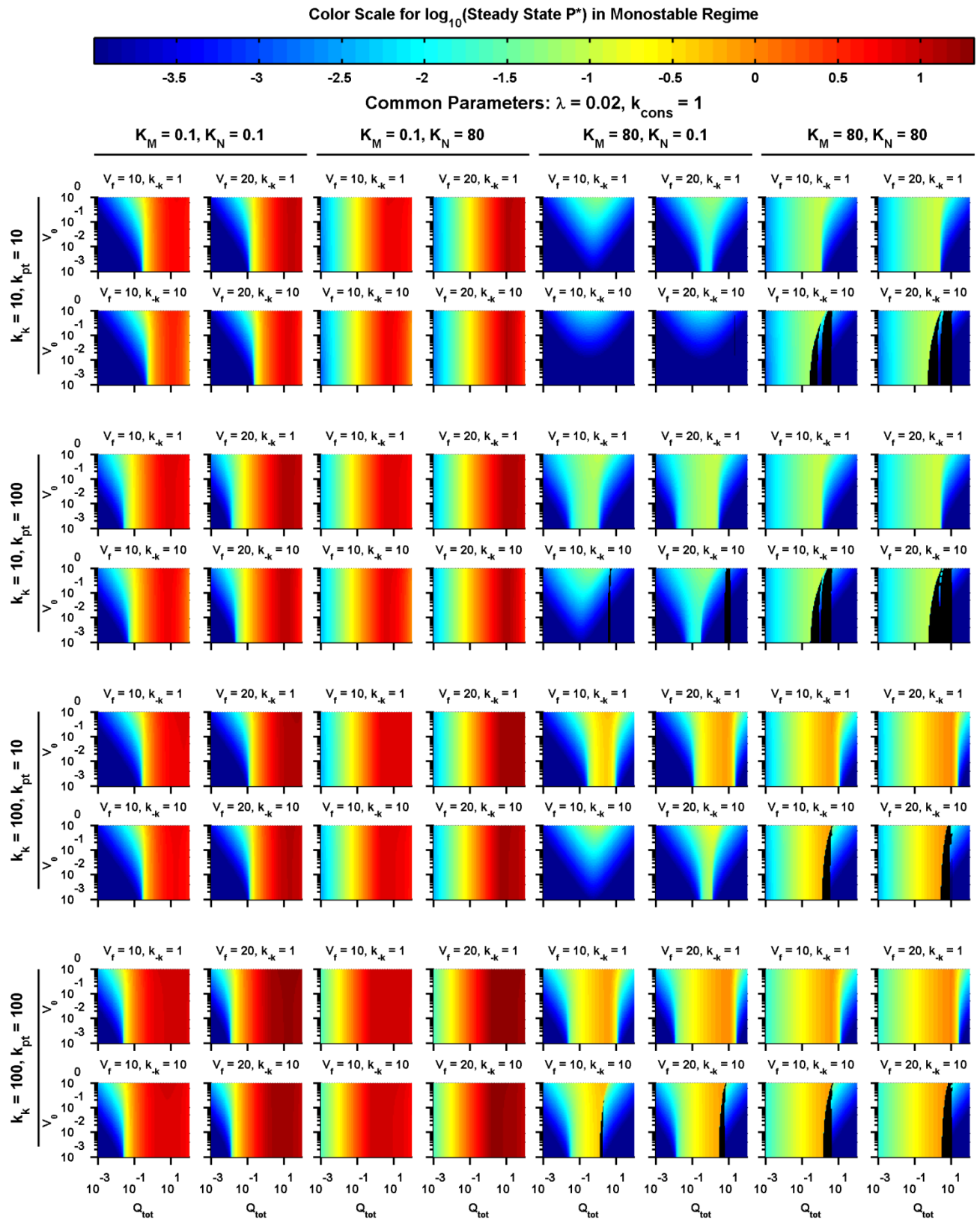

Supplement: Figure S9 — Region of bistability in a model where phoQ (T281R) expression is decoupled from PhoP-P control. Steady state behavior of a modified PhoQ/PhoP network with autoregulated phoP and constitutive phoQ (T281R) was analyzed (Text S1) to determine parameter values for which the network showed bistability. The bistable regime is plotted in black. In the monostable regime, the steady state value of P* is plotted according to the color scale on the top. Steady state values less than 10−4 are plotted as 10−4. The figure is divided into an 8×8 grid of panels. In each panel, the total concentration of PhoQ (Qtot) is varied along the x-axis, whereas the constitutive promoter strength (V0) is varied along the y-axis. λ = 0.02 and kcons = 1 for all panels. Parameters common to each pair of rows (or columns) are indicated on the left (or top). The remaining parameter values are indicated on the top of each panel. See Text S1 for definitions of parameters. (PDF) [file pgen.1003706.s009.pdf]
