# Supplementary material for: The Architecture of a Prototypical Bacterial Signaling Circuit Enables a Single Point Mutation to Confer Novel Network Properties
Source: PLoS Genet. 2013 Aug 22;9(8):e1003706. doi: 10.1371/journal.pgen.1003706 (PMC3750022; doi:10.1371/journal.pgen.1003706)
Supplement: Table S1 — List of strains. (PDF) [file pgen.1003706.s010.pdf]

Table S1. List of Strains

Strain	Relevant Genotype	Source or Reference
BW23473	Host strain for CRIM plasmid excision	[1]
JW1115	BW25113 Δ(*phoQ*)::*kan*	[2]
MG1655		*E. coli* Genetic Stock Center, CGSC no. 7740
SRI027	MG1655 Δ*phoPQ* $att\phi_{80}$::[pTM168]	This study
SRI030	MG1655 Δ*lacZYA* Δ(*phoQ*)::*kan* *attλ*::[pTM79 Δ*cat*] *attHK*::[pTM27 Δ*kan*]	This study
SRI035	MG1655 Δ*phoPQ* $att\phi_{80}$::[pTM168 Δ(*phoP*)::*kan*]	This study
SRI038	MG1655 Δ*lacZYA* Δ*phoQ* *attλ*::[pTM79 Δ*cat*] *attHK*::[pTM27 Δ*kan*]	This study
SRI040	MG1655 Δ*phoPQ* $att\phi_{80}$::[pTM168 Δ*phoP*]	This study
SRI043	MG1655 *attλ*::[pTM83 Δ(*cat*-P_2)::*kan*][1]	This study
SRI046	MG1655 Δ*lacZYA* Δ*phoQ* *attλ*::[pTM79 Δ*cat*] *attHK*::[pTM27 Δ*kan*] $att\phi_{80}$::[pSR014]	This study
SRI049	MG1655 Δ*phoPQ* Δ*lacZYA* *attλ*::[pTM83 Δ(*cat*-P_2)::*kan*] *attHK*::[pTM27 Δ*kan*][1]	This study
SRI054	MG1655 ΔphoQ::*cat*-<IsceI>[2]	This study
SRI055	MG1655 ΔphoQ::*cat*-<IsceI> *attλ*::[pTM79 Δ*cat*] *attHK*::[pTM27 Δ*kan*][2]	This study
SRI056	MG1655 *attλ*::[pTM83 Δ(*cat*)::*kan*]	This study
SRI057	MG1655 Δ*phoPQ* Δ*lacZYA* *attλ*::[pTM83 Δ(*cat*)::*kan*] *attHK*::[pTM27 Δ*kan*]	This study
SRI058	MG1655 *phoQ (T281R)* *attλ*::[pTM79 Δ*cat*] *attHK*::[pTM27 Δ*kan*]	This study
SRI059	MG1655 Δ*phoPQ* Δ*lacZYA* *attλ*::[pTM83 Δ(*cat*-P_2)::*kan*] *attHK*::[pTM27 Δ*kan*] $att\phi_{80}$::[pTM168][1]	This study
SRI060	MG1655 Δ*phoPQ* Δ*lacZYA* *attλ*::[pTM83 Δ(*cat*)::*kan*] *attHK*::[pTM27 Δ*kan*] $att\phi_{80}$::[pTM168]	This study
SRI066	MG1655 *phoQ (T281R)*	This study
SRI069	MG1655 *phoQ (T281R)* Δ(P_2)::*kan*[1]	This study
SRI073	MG1655 *phoQ (T281R)* Δ(P_2)::*kan* *attλ*::[pTM79 Δ*cat*] *attHK*::[pTM27 Δ*kan*][1]	This study
SRI078	MG1655 *phoQ (T281R)* *attλ*::[pTM79 Δ*cat*] *attHK*::[pTM27 Δ(*kan)::cat*]	This study
TIM13	MG1655 Δ*phoPQ*	[3]
TIM61	MG1655 *attHK*::[pTM27 Δ(*kan)::cat*]	T. Miyashiro and M.G., unpublished
TIM92	MG1655 *attλ*::[pTM79 Δ*cat*] *attHK*::[pTM27 Δ*kan*]	[4]
TIM147	MG1655 *attλ*::[pTM83]	T. Miyashiro and M.G., unpublished
TIM176	MG1655 Δ*phoPQ* Δ*lacZYA* *attλ*::[pTM83] *attHK*::[pTM27 Δ*kan*]	[4]
TIM210	MG1655 Δ*lacZYA* *attλ*::[pTM79 Δ*cat*] *attHK*::[pTM27 Δ*kan*]	[3]

References

1. Haldimann A, Wanner BL (2001) Conditional-replication, integration, excision, and retrieval plasmid-host systems for gene structure-function studies of bacteria. J Bacteriol 183: 6384-6393.
2. Baba T, Ara T, Hasegawa M, Takai Y, Okumura Y, et al. (2006) Construction of Escherichia coli K-12 in-frame, single-gene knockout mutants: the Keio collection. Mol Syst Biol 2: 2006 0008.
3. Miyashiro T, Goulian M (2008) High stimulus unmasks positive feedback in an autoregulated bacterial signaling circuit. Proceedings of the National Academy of Sciences of the United States of America 105: 17457-17462.
4. Miyashiro T, Goulian M (2007) Stimulus-dependent differential regulation in the Escherichia coli PhoQ-PhoP system. Proceedings of the National Academy of Sciences of the United States of America 104: 16305-16310.

[1] P_2 refers to the constitutive promoter of the *phoPQ* operon.

[2] <IsceI> is the recognition site for the endonuclease I-SceI.
