# Supplementary material for: The Architecture of a Prototypical Bacterial Signaling Circuit Enables a Single Point Mutation to Confer Novel Network Properties
Source: PLoS Genet. 2013 Aug 22;9(8):e1003706. doi: 10.1371/journal.pgen.1003706 (PMC3750022; doi:10.1371/journal.pgen.1003706)
Supplement: Table S2 — List of plasmids. (PDF) [file pgen.1003706.s011.pdf]

Table S2. List of Plasmids

Plasmid	Relevant Genotype	Source or Reference
pAH123	λ*cI857*(ts) *repA101*(ts) *oriR101 bla* λp_{R}-$int_{\varphi 80}$	[1]
pAH129	λ*cI857*(ts) *repA101*(ts) *oriR101 bla* λp_{R}- ($xis_{\varphi 80}$ $int_{\varphi 80}$)	[1]
pCP20	λ*cI857*(ts) *repA101*(ts) *oriR101 bla cat* λp_{R}-FLP	[2]
pKD4	$oriR_{\gamma R6k}$ *bla FRT*::*kan*::*FRT*	[3]
pKD13	$oriR_{\gamma R6k}$ *bla FRT*::*kan*::*FRT*	[3]
pKD46	*repA101*(ts) *oriR101 bla* P_{araB}-(*gam bet exo*)	[3]
pSR014	pTM168 *ΔphoP*	This work
pTM27	$oriR_{\gamma R6k}$ *kan* $attP_{HK}$ P_{tetA}-*cfp*	[4]
pTM79	$oriR_{\gamma R6k}$ *cat* $attP_{\lambda}$ P_{mgrB}-*yfp*	[4]
pTM83	$oriR_{\gamma R6k}$ *cat* $attP_{\lambda}$ P_{phoPQ}-*yfp*	[4]
pTM168	$oriR_{\gamma R6k}$ *cat* $attP_{\varphi 80}$ $lacI^{q}$ P_{trc}-*(phoP-phoQ(T281R))*	[5]
pTM177	$oriR_{\gamma R6k}$ *cat* $attP_{\varphi 80}$ $lacI^{q}$ P_{phoPQ}-*(phoP-phoQ(T281R))*	[5]
pWRG99	pKD46 P_{tet}-(*I-SceI tetR*)	[6]
pWRG100	$oriR_{\gamma R6k}$ *cat*-<IsceI>[1]	[6]

References

1. Haldimann A, Wanner BL (2001) Conditional-replication, integration, excision, and retrieval plasmid-host systems for gene structure-function studies of bacteria. J Bacteriol 183: 6384-6393.
2. Cherepanov PP, Wackernagel W (1995) Gene disruption in Escherichia coli: TcR and KmR cassettes with the option of Flp-catalyzed excision of the antibiotic-resistance determinant. Gene 158: 9-14.
3. Datsenko KA, Wanner BL (2000) One-step inactivation of chromosomal genes in Escherichia coli K-12 using PCR products. Proc Natl Acad Sci U S A 97: 6640-6645.
4. Miyashiro T, Goulian M (2007) Stimulus-dependent differential regulation in the Escherichia coli PhoQ-PhoP system. Proceedings of the National Academy of Sciences of the United States of America 104: 16305-16310.
5. Miyashiro T, Goulian M (2008) High stimulus unmasks positive feedback in an autoregulated bacterial signaling circuit. Proceedings of the National Academy of Sciences of the United States of America 105: 17457-17462.
6. Blank K, Hensel M, Gerlach RG (2011) Rapid and highly efficient method for scarless mutagenesis within the Salmonella enterica chromosome. PLoS One 6: e15763.

[1] <IsceI> is the recognition site for the endonuclease I-SceI.
