# Supplementary material for: The Architecture of a Prototypical Bacterial Signaling Circuit Enables a Single Point Mutation to Confer Novel Network Properties
Source: PLoS Genet. 2013 Aug 22;9(8):e1003706. doi: 10.1371/journal.pgen.1003706 (PMC3750022; doi:10.1371/journal.pgen.1003706)
Supplement: Table S3 — List of primers. (PDF) [file pgen.1003706.s012.pdf]

Table S3. List of Primers

Primer	Sequence
P1	**GTGATTACCACCGTTCGCGGCCAGGGCTATCTGTTCGAATTGCGCTGATGCGCCTTACGCCCCGCCCTGC**
P2	**TTACCCTCATATTGCTCGGTGATTTCGCGGGCTACCGCCAGCCCTACACCCTAGACTATATTACCCTGTT**
P3	**TGTACTGATGGGACGTCTGC**
P4	**GCGTGAAGTATGGGCATATT**
P5	**CTAACTATATTGGTCGAGCTATCACGATGGTTGATGAGCTGAAATAAACCATGAATATCCTCCTTAG**
P6	**CGATTATGTGGTTATGGGGGTAAACATTAAATAAACCAGCGGGGAGGGGAGCTGGAGCTGCTTCGAA**
P7	**CGGGGATCCTCTAGAACTAGTGGATCCCCCGACAGGGAGAAATAAAAATGATTCCGGGGATCCGTCGACC**
P8	**CGGGAAAAAAAGACGCAGTAATTTTTTCATCAGCGCAATTCGAACAGATATGTAGGCTGGAGCTGCTTCG**
P9	**ATGAACCTGAATCGCCAGCGGCATCAGCACCTTGTCGCCTTGCGTATAATATGAATATCCTCCTTAG**
P10	**ATATCCCAATGGCATCGTAAAGAACATTTTGAGGCATTTCAGTCAGTTGCGCTGGAGCTGCTTCGAA**
