# Supplementary material for: The Architecture of a Prototypical Bacterial Signaling Circuit Enables a Single Point Mutation to Confer Novel Network Properties
Source: PLoS Genet. 2013 Aug 22;9(8):e1003706. doi: 10.1371/journal.pgen.1003706 (PMC3750022; doi:10.1371/journal.pgen.1003706)
Supplement: Table S4 — Figure-wise list of strains. (PDF) [file pgen.1003706.s013.pdf]

Table S4. Figure-wise List of Strains

Figure	Strain Name	Strain Designation in Figure
**1**	TIM92	*phoQ (WT)*
	SRI058	Strain depicted in panel A. *phoQ (T281R)* in panel B. *phoQ (T281R)* OFF (YFP-dim colony) and *phoQ (T281R)* ON (YFP-bright colony) in panels C and D.
**2**	SRI058	Strain used for data presented in panels B-D
**4**	SRI058	T281R OFF Cm^S (YFP-dim colony) and T281R ON Cm^S (YFP-bright colony)
	SRI078	T281R OFF Cm^R (YFP-dim colony) and T281R ON Cm^R (YFP-bright colony)
**5**	SRI058	*phoQ (T281R)* OFF (YFP-dim colony) and *phoQ (T281R)* ON (YFP-bright colony)
**6**	SRI046	Strain depicted in panel A, and used for data presented in panel B
	SRI058	*phoQ (T281R)* OFF in panel D
	SRI073	Strain depicted in panel C. ΔP_2 OFF (YFP-dim colony) and ΔP_2 ON (YFP-bright colony) in panel D
**S6**	SRI059	ΔP_2 Reporter Strain
	SRI060	*phoPQ* Reporter Strain
