# Supplementary material for: The Architecture of a Prototypical Bacterial Signaling Circuit Enables a Single Point Mutation to Confer Novel Network Properties
Source: PLoS Genet. 2013 Aug 22;9(8):e1003706. doi: 10.1371/journal.pgen.1003706 (PMC3750022; doi:10.1371/journal.pgen.1003706)
Supplement: Table S5 — Parameters and reactions for simulation of the autoregulated stochastic model. (PDF) [file pgen.1003706.s014.pdf]

Table S5. Parameters and Reactions for Simulation of the Autoregulated Stochastic Model

**Molecular Species**			
PQrna	mRNA encoding for PhoP and PhoQ		
P	PhoP protein		
Q	PhoQ protein		
P*	PhoP-P		
Q*	PhoQ-P protein		
PQ*	Complex between PhoP and PhoQ-P		
P*Q	Complex between PhoP-P and PhoQ		
**Network Reactions**			
	**Reaction**	**Propensity Function**	**Parameter Values**
1	P2 Promoter → PQrna	C_{P2}	$C_{P2} = 0.3\ s^{-1}$ for normal promoter<br>$C_{P2} = 0.03\ s^{-1}$ for ΔP_2 promoter
2	P1 Promoter → PQrna	C_{P1}P*/(K*+P*)	$C_{P1} = 5, 7.5, 12.5, 20\ s^{-1}$ corresponding to Parameter Sets 1-4 in Fig. S5A<br>K*=500
3	PQrna → P	k_PPQrna	$k_P = 10^{-2}\ s^{-1}$
4	PQrna → Q	k_QPQrna	$k_Q = 2 \times 10^{-4}\ s^{-1}$
5	P + Q* → PQ*	k_5P×Q*	$k_5 = 2 \times 10^{-3}\ s^{-1}$
6	PQ* → P + Q*	k_6PQ*	$k_6 = 10\ s^{-1}$
7	P* + Q → P*Q	k_7P*×Q	$k_7 = 2 \times 10^{-3}\ s^{-1}$
8	P*Q → P* + Q	k_8P*Q	$k_8 = 10\ s^{-1}$
9	Q → Q*	k_9Q	$k_9 = 5 \times 10^{-2} s^{-1}$
10	Q* → Q	k_{10}Q*	$k_{10} = 5 \times 10^{-3}\ s^{-1}$
11	PQ* → P* + Q	k_{11}PQ*	$k_{11} = 5 \times 10^{-2} s^{-1}$
12	P → Ø	kP	
13	Q → Ø	kQ	k =Growth Rate
14	P* → Ø	kP*	$k = 2.5 \times 10^{-4}\ s^{-1}$ for slow growth rate
15	Q* → Ø	kQ*	
16	PQ* → Ø	kPQ*	$k = 5 \times 10^{-4}\ s^{-1}$ otherwise
17	P*Q → Ø	kP*Q	
18	PQrna → Ø	k_{RNA}PQrna	$k_{RNA} = 5 \times 10^{-2}\ s^{-1}$

**Initial Conditions**		
**Species**	**OFF state**	**ON State**
PQrna	2	71
P	2	1029
Q	2	6
P*	2	373
Q*	2	19
PQ*	2	4
P*Q	2	1
