# Supplementary material for: The Architecture of a Prototypical Bacterial Signaling Circuit Enables a Single Point Mutation to Confer Novel Network Properties
Source: PLoS Genet. 2013 Aug 22;9(8):e1003706. doi: 10.1371/journal.pgen.1003706 (PMC3750022; doi:10.1371/journal.pgen.1003706)
Supplement: Table S6 — Parameters and reactions for simulation of the decoupled stochastic model. (PDF) [file pgen.1003706.s015.pdf]

Table S6. Parameters and Reactions for Simulation of the Decoupled Stochastic Model

**Molecular Species**			
Prna	mRNA encoding for PhoP		
Qrna	mRNA encoding for PhoQ		
P	PhoP protein		
Q	PhoQ protein		
P*	PhoP-P		
Q*	PhoQ-P protein		
PQ*	Complex between PhoP and PhoQ-P		
P*Q	Complex between PhoP-P and PhoQ		
**Network Reactions**			
	**Reaction**	**Propensity Function**	**Parameter Values**
1	P2 Promoter → Prna	C_{P2}	$C_{P2} = 0.3\ s^{-1}$
2	P1 Promoter → Prna	C_{P1}P*/(K*+P*)	$C_{P1} = 7.5\ s^{-1}$<br>K*=500
3	Q Promoter → Qrna	C_Q	C_Q indicated in Fig. S5D
4	Prna → P	k_PPrna	$k_P = 10^{-2}\ s^{-1}$
5	Qrna → Q	k_QQrna	$k_Q = 2 \times 10^{-4}\ s^{-1}$
6	P + Q* → PQ*	k_5P×Q*	$k_5 = 2 \times 10^{-3}\ s^{-1}$
7	PQ* → P + Q*	k_6PQ*	$k_6 = 10\ s^{-1}$
8	P* + Q → P*Q	k_7P*×Q	$k_7 = 2 \times 10^{-3}\ s^{-1}$
9	P*Q → P* + Q	k_8P*Q	$k_8 = 10\ s^{-1}$
10	Q → Q*	k_9Q	$k_9 = 5 \times 10^{-2} s^{-1}$
11	Q* → Q	k_{10}Q*	$k_{10} = 5 \times 10^{-3}\ s^{-1}$
12	PQ* → P* + Q	k_{11}PQ*	$k_{11} = 5 \times 10^{-2} s^{-1}$
13	P → Ø	kP	
14	Q → Ø	kQ	
15	P* → Ø	kP*	k =Growth Rate
16	Q* → Ø	kQ*	$k = 5 \times 10^{-4}\ s^{-1}$
17	PQ* → Ø	kPQ*	
18	P*Q → Ø	kP*Q	
19	Prna → Ø	k_{RNA}Prna	$k_{RNA} = 5 \times 10^{-2}\ s^{-1}$
20	Qrna → Ø	k_{RNA}Qrna	$k_{RNA} = 5 \times 10^{-2}\ s^{-1}$

**Initial Conditions**		
**Species**	**Uninduced State**	**Induced State**
Prna	2	121
Qrna	2	400
P	2	766
Q	2	29
P*	2	1623
Q*	2	107
PQ*	2	17
P*Q	2	10
