# Supplementary material for: The Architecture of a Prototypical Bacterial Signaling Circuit Enables a Single Point Mutation to Confer Novel Network Properties
Source: PLoS Genet. 2013 Aug 22;9(8):e1003706. doi: 10.1371/journal.pgen.1003706 (PMC3750022; doi:10.1371/journal.pgen.1003706)
Supplement: Text S1 — Modeling the PhoQ/PhoP network. (PDF) [file pgen.1003706.s016.pdf]

Text S1. Modeling of the PhoQ/PhoP Network

A. Simple Model of the PhoQ/PhoP Network

We first describe a simple model of the PhoQ/PhoP network that compares *phoQ (WT)* and *phoQ (T281R)* and recapitulates many of the experimentally observed properties of the system. This model is referred to in Figures S1A and S7.

We model the PhoQ/PhoP network as consisting of three species: P, P* and Q. P and P* play the role of PhoP and PhoP-P respectively, whereas bifunctional PhoQ is represented as Q. The phosphorylation state of PhoQ is ignored in this model. Transcription and translation of the *phoPQ* operon are modeled as a single step. Production rate of P is assumed to be a sum of contributions from the constitutive P_2 promoter and the P* responsive P_1 promoter as follows:

$$V_0' + V_f' \frac{[P^*]}{K^* + [P^*]}$$

where V_0' parameterizes the constitutive production, and V_f' and K^* parameterize the feedback from P*. K^* is the concentration of P* at which feedback-mediated production rate of P is half-maximal. We define $[P_{tot}]$ and $[Q_{tot}]$ to be the total concentration of PhoP and PhoQ in the system. In this model, $[P_{tot}] = [P] + [P^*]$ and $[Q_{tot}] = [Q]$. Since *phoP* and *phoQ* are in an operon driven by a common promoter, $[P_{tot}]$ and $[Q_{tot}]$ are assumed to be proportional:

$$[Q_{tot}] = \lambda [P_{tot}]$$

The production of P* from P is modeled as a bimolecular reaction between P and Q with rate constant k_k'. Similarly, the phosphatase reaction is modeled as bimolecular reaction between P* and Q with rate constant k_p'. Finally, we assume that all three species are stable proteins and that their concentrations are affected by dilution through growth at a rate equivalent to the growth rate k.

We choose $[P^*]$, $[P_{tot}]$ and $[Q_{tot}]$ to be the state variables. At steady state, the production of P* from P must be balanced by the phosphatase flux and the dilution of P* through growth:

$$k_k'[P][Q] = k_p'[P^*][Q] + k[P^*] \qquad (1)$$

This can be rewritten as:

$$k_k'([P_{tot}] - [P^*])[Q_{tot}] = k_p'[P^*][Q_{tot}] + k[P^*] \qquad (2)$$

At steady state, the production of P must be balanced by dilution of P and P* through growth:

$$V_0' + V_f' \frac{[P^*]}{K^* + [P^*]} = k[P_{tot}] \qquad (3)$$

The third constraint required to obtain the steady state is:

$$[Q_{tot}] = \lambda [P_{tot}] \qquad (4)$$

The number of parameters can be reduced by transforming to dimensionless variables and parameters by scaling all concentrations to K^* and all rates to k as follows:

$$P_{tot} = \frac{[P_{tot}]}{K^*}, Q_{tot} = \frac{[Q_{tot}]}{K^*}, P^* = \frac{[P^*]}{K^*}$$
$$k_k = \frac{k'_k K^*}{k}, k_p = \frac{k'_p K^*}{k}, V_0 = \frac{V'_0}{kK^*}, V_f = \frac{V'_f}{kK^*}$$

Then, equations (2)-(4) transform to:

$$k_k(P_{tot} - P^*)Q_{tot} = k_p P^* Q_{tot} + P^* \quad (5)$$
$$V_0 + V_f \frac{P^*}{1 + P^*} = P_{tot} \quad (6)$$
$$Q_{tot} = \lambda P_{tot} \quad (7)$$

Substituting the expressions for P_{tot} and Q_{tot} from equations (6) and (7) into equation (5) yields a cubic equation in P* whose roots represent potential steady states of the system:

$$\begin{gathered}(-\lambda k_t V - 1)(P^*)^3 + (\lambda k_k V^2 - \lambda k_t V - \lambda k_t V_0 - 2)(P^*)^2 \\ +(2\lambda k_k V_0 V - \lambda k_t V_0 - 1)P^* + \lambda k_k V_0^2 = 0\end{gathered} \quad (8)$$

where $V \equiv V_0 + V_f$ and $k_t \equiv k_k + k_p$.

For bistability, equation (8) must have 3 non-negative roots. A systematic exploration of parameters reveals that the network readily shows bistability especially at low V_0 values (Figure S7). However, at a given V_0, the region of bistability shrinks as the kinase rate k_k increases. As will be discussed below, there are two non-linearities in the system that are essential for bistability: (1) the second-degree terms that quantify the phosphatase and kinase reaction rates ($k_k(P_{tot} - P^*)Q_{tot}$ and $k_p P^* Q_{tot}$ respectively), and (2) the non-linear dependence (in particular, saturation) of the expression of the *phoPQ* operon on P* concentration.

To explain the difference between *phoQ (WT)* and *phoQ (T281R)*, we need to consider that the *phoQ (T281R)* mutant is not only phosphatase-deficient, but also a poorer kinase [1]. This would correspond to a lower value of the parameter k_k for *phoQ (T281R)* compared to *phoQ (WT)*. Bistability is not seen in *phoQ (WT)* for one of three reasons:

(1) The kinase rate is high enough that for all values of the phosphatase rate, the system is monostable. This scenario is depicted in Figure S1A (left panel) and contrasted with the situation in *phoQ (T281R)* where the lower kinase rate makes bistability possible at low phosphatase rates (Figure S1A, right panel). The following parameters were used to plot Figure S1A: $\lambda = 0.02, V_0 = 0.1, V_f = 10$. In addition, $k_k = 50$ for the left panel and $k_k = 10$ for the right panel. Steady state P* values for these parameters were computed numerically using equation (8) at different values of k_p and plotted as a function of k_p. Comparison of the steady states in the high kinase, high phosphatase (*phoQ (WT)*) and low kinase, low phosphatase (*phoQ (T281R)*) cases in Figure S1A shows that the WT steady state can be in between the low and high values of T281R consistent with experimental results depicted in Figure 1D.

(2) The kinase rate is low enough that at low phosphatase rates the system could show bistability. However, such low kinase rates are associated with high phosphatase rates in *phoQ (WT)* and the predominant mechanism for loss of P* under these circumstances is the phosphatase action

of PhoQ (dilution of P* due to growth can be neglected). In this limit, the network can be shown to be monostable as discussed below.

(3) The kinase rate and phosphatase rates are such that it is not possible to achieve P* levels high enough to begin to saturate the P* dependent feedback expression of the operon. In this scenario, the network cannot be bistable as will be discussed below. It should be noted that in wild-type *E. coli*, this scenario holds at a Mg^{2+} concentration of 100µM, which is considered an activating stimulus [2].

Sufficient Criteria For Monostability

1. Phosphatase Flux Dominates Over Dilution Flux of P*

In this scenario, the phosphatase rate is assumed to be high enough that the predominant mechanism for the loss of P* from the system is the phosphatase reaction. Mathematically, this condition can be stated as $k_p Q_{tot} \gg 1$. The loss of P* by dilution due to growth can be neglected. In this case, equation (5) can be simplified to the following:

$$k_k(P_{tot} - P^*) = k_p P^* \quad (9)$$

Substituting the expression for P_{tot} from equation (6) yields a quadratic equation in P* whose roots represent potential steady states of the system:

$$-k_t(P^*)^2 + (k_k V - k_t)P^* + k_k V_0 = 0 \quad (10)$$

This equation has one positive root, and thus the system is monostable.

2. Decoupling of expression of Q from P* regulation

The second-degree non-linearity in the phosphatase and kinase reaction rates ($k_k(P_{tot} - P^*)Q_{tot}$ and $k_p P^* Q_{tot}$ respectively) can be eliminated in an altered *phoPQ* network where the expression of P remains P* sensitive, but expression of Q is constitutive (i.e. insensitive to P* levels). In this case, Q_{tot} is a constant determined by the constitutive expression rate of Q and the kinase and phosphatase reaction rates become linear in P and P* respectively. Equations (5) and (6) are now sufficient to determine steady state P* levels. Substituting the expression for P_{tot} from equation (6) into equation (5) yields a quadratic equation in P* whose roots represent potential steady states of the system:

$$-(k_t Q_{tot} + 1)\,(P^*)^2 + (k_k V Q_{tot} - k_t Q_{tot} - 1)P^* + k_k Q_{tot} V_0 = 0 \quad (11)$$

This equation also has one positive root, and thus the system is again monostable.

3. P* levels sufficiently low that feedback expression of *phoPQ* operon depends linearly on P* concentrations

In general, the P* dependent feedback expression of the *phoPQ* operon is a non-linear function of P* concentration. However, at low levels of P*, the function can be approximated by a linear

function. Mathematically, this regime is characterized by the constraint that $[P^*] \ll K^*$, or $P^* \ll 1$ in terms of the scaled variable. In this regime, equation (6) can be simplified to:

$$V_0 + V_f P^* = P_{tot} \tag{12}$$

Substituting the expressions for P_{tot} and Q_{tot} from equations (12) and (7) into equation (5) yields a quadratic equation in P* whose roots represent potential steady states of the system:

$$\lambda V_f (k_k V_f - k_t)(P^*)^2 + (2\lambda k_k V_0 V_f - \lambda k_t V_0 - 1)P^* + \lambda k_k V_0^2 = 0 \tag{13}$$

where $k_t \equiv k_k + k_p$. Equation (13) can have at most two non-negative roots, and only one of these can be a stable state. However, it is possible for equation (13) to have no non-negative roots. In that case, P* steadily increases and the constraint $P^* \ll 1$ is violated. Thus, if the network parameters are such that non-linear nature of the dependence of *phoPQ* operon expression on P* is never encountered, the network is always monostable.

<u>Monostable and Bistable Regimes in the Phosphatase-deficient Mutant</u>

In the phosphatase-deficient mutant $(k_p = 0)$, two parameters largely determine the nature of roots of equation (8). These are the kinase rate (k_k) and the maximal operon expression rate (V). If either the kinase rate or the maximal operon expression rate is zero, then $P^* = 0$ is the only non-negative solution to equation (8). As shown in Figure S7, for sufficiently high values of k_k , V_0 or V_f, the system is monostable with a high value of P* $(P^* > 1)$. Thus, starting from a set of parameters for which the system is bistable, one should enter a monostable regime with a low or high steady state P* value if one decreased or increased k_k (or V) sufficiently while keeping other parameters constant. Since all kinetic parameters are scaled to the growth rate, an increase in k_k or V can be achieved either by increasing the unscaled kinase rate (k_k') , the unscaled maximal operon expression rate $(V_0' + V_f')$ or by decreasing the growth rate (k). The qualitative effect of varying these parameters is depicted in Figure 3B (Higher Mg^{2+} concentrations are assumed to increase either the unscaled kinase rate or the unscaled maximal operon expression rate in the *phoQ (T281R)* network).

B. Detailed Model of the *phoQ (T281R)* mutant

This model is an extension of the *phoQ (WT)* model described previously [1] to the case of zero phosphatase rate. Key assumptions and parameter definitions are stated below. The actual algebraic manipulations were performed using MATLAB's Symbolic Math Toolbox and code is available upon request.

The model consists of six species: P, P*, Q, Q*, P*Q and PQ*. P, P*, Q and Q* represent PhoP, PhoP-P (phosphorylated PhoP), PhoQ and PhoQ-P (phosphorylated PhoQ) respectively. PQ* is a complex between P and Q* and is an intermediate state in the phosphotransfer step. P*Q is a complex between P* and Q and is an intermediate state in the phosphatase reaction. In the phosphatase mutant, P*Q is taken to be a dead-end complex. Note that this dead-end complex is different from

the dead-end complex between P and Q considered in reference [3] as a potential source of bistability in two-component systems.

Binding and unbinding reactions for the formation of complex P*Q are assumed to be fast and in equilibrium. As in the original model, ATP is not included explicitly. ATP concentration in the cell is assumed to be constant and this concentration is absorbed into the kinetic constant for the autophosphorylation reaction of *phoQ (T281R)*.

We now describe the post-translational reactions that are included in the model and the parameters associated with them:

1. Autophosphorylation and Dephosphorylation of Q with rate constants k'_k and k'_{-k} respectively:
$$Q \underset{k'_k}{\rightarrow} Q^*, Q^* \underset{k'_{-k}}{\longrightarrow} Q$$

2. Phosphotransfer from Q* to P
$$Q^* + P \underset{k_r^{PQ^*}}{\overset{k_f^{PQ^*}}{\rightleftarrows}} PQ^* \underset{k'_{pt}}{\rightarrow} P^* + Q$$

   Here $k_f^{PQ^*}$ and $k_r^{PQ^*}$ are the rate constants for the association and dissociation of the PQ* complex respectively and k'_{pt} is the rate constant for the phosphotransfer step.

3. Formation of Dead-end Complex between P* and Q
$$Q + P^* \underset{K_d^{P^*Q}}{\leftrightharpoons} P^*Q$$

   Here $K_d^{P^*Q}$ is the equilibrium dissociation constant for the P*Q complex.

4. Conversion of P* to P through spontaneous hydrolysis or constitutive non-specific phosphatase with a first-order rate constant k'_{cons}.
$$P^* \underset{k'_{cons}}{\longrightarrow} P$$

Autoregulation of the *phoPQ* operon is modeled as a single transcription/translation step. Production rate of P is assumed to be a sum of contributions from the constitutive P_2 promoter and the P* responsive P_1 promoter as follows:
$$V'_0 + V'_f \frac{[P^*]}{K^* + [P^*]}$$
where V'_0 parameterizes the constitutive production, and V'_f and K^* parameterize the feedback from P*. K^* is the concentration of P* at which feedback-mediated production of P is half-maximal.

Since *phoP* and *phoQ* are in an operon driven by a common promoter, the production rate of Q is assumed to be proportional to that of P:

$$\lambda\left(V_0' + V_f' \frac{[P^*]}{K^* + [P^*]}\right)$$

where λ is the constant of proportionality.

All six species are assumed to be stable proteins/complexes whose concentrations are affected by dilution through growth at a rate equivalent to the growth rate k.

As in the simple model described above, we transform to dimensionless variables and parameters by scaling all concentrations to K^* and all rates to k. The scaled concentrations of the six species P, P*, Q, Q*, P*Q and PQ* are represented by variables P, P*, Q, Q*, $C^{P^*Q}$and C^{PQ^*} respectively. We also define P_{tot} and Q_{tot} to be the total scaled concentration of PhoP-species and PhoQ-species respectively. We now present the steady-state determining equations in terms of these scaled variables and parameters.

The equilibrium assumption for P*Q and the steady state condition for PQ* yield two constraints:

$$C^{P^*Q} = K_M Q P^* \quad (14)$$

$$C^{PQ^*} = K_N P Q^* \quad (15)$$

where $K_M \equiv \frac{K^*}{K_d^{P^*Q}}$ and $K_N \equiv \frac{K^* k_f^{PQ^*}}{k_r^{PQ^*} + k'_{pt} + k}$.

At steady state, production and loss of Q* and P*must balance:

$$k_k Q = k_{pt} C^{PQ^*} + \ k_{-k} Q^* \quad (16)$$

$$k_{pt} C^{PQ^*} = C^{P^*Q} + (1 + k_{cons}) P^* \quad (17)$$

where $k_k \equiv \frac{k'_k}{k}, k_{pt} \ \equiv \frac{k'_{pt}}{k}, k_{-k} \ \equiv 1 + \frac{k'_{-k}}{k},$ and $k_{cons} \ \equiv \frac{k'_{cons}}{k}$. Also, at steady state, the production of P must be balanced by dilution through growth of all PhoP-species:

$$V_0 + V_f \frac{P^*}{1 + P^*} = P_{tot} \quad (18)$$

where $V_0 \equiv \frac{V'_0}{kK^*}$ and $V_f \ \equiv \frac{V'_f}{kK^*}$.

By definition, the total concentration of PhoQ-species is:

$$Q_{tot} = Q + Q^* + C^{PQ^*} + C^{P^*Q} \quad (19)$$

We now use the experimentally-observed fact that $Q_{tot} \ll P_{tot}$ (i.e. $\lambda \ll 1$) to approximate P_{tot} as:

$$P_{tot} = P + P^* \quad (20)$$

The final constraint is that of the proportionality of P_{tot} and Q_{tot} , namely:

$$Q_{tot} = \lambda P_{tot} \quad (21)$$

Equations (14)-(21) define 8 constraints in 8 variables (6 species, P_{tot} , Q_{tot}). These can be used to obtain a quintic equation in P* whose roots represent potential steady states of the system:

$$\sum_{i=0}^{5} a_i \, (P^*)^i = 0 \quad (22)$$

Depending on parameter values, equation (22) can either have 2 or 4 positive real roots (one root is always negative provided $V_0 \neq 0$), which correspond to monostable and bistable regimes. A systematic exploration of parameters was performed to determine the bistable regime (Figure S8). In Figure S8, we can see that whenever the parameters allow a stable state where the non-linear nature of the transcriptional feedback becomes relevant ($P^* > 1$, or equivalently $[P^*] > K^*$) , one can obtain a parameter set demonstrating bistability by reducing V_0while keeping all other parameters same. On the other hand, bistability is lost if V_0 is increased sufficiently. This effect is depicted in Figure S1B. The following parameters were used to plot Figure S1B: $\lambda = 0.02, V_f = 20, K_M = 0.1, K_N = 0.1, k_{pt} = 100, k_k = 100, k_{-k} = 10$, and $k_{cons} = 1$. Steady state P* values for these parameters were computed numerically using equation (22) at different values of V_0 and plotted as a function of V_0.

Equation (22) also allows us to look at the effect of changes in growth rate on steady state values of P*. We assume that changes in growth rate do not affect the unscaled kinetic parameters – these are assumed to be intrinsic parameters of the proteins or the *phoPQ* promoter. In the scaled model, however, since the growth rate is set to 1, a reduction (or increase) in true growth rate is equivalent to upscaling (or downscaling) of all other parameters as follows:

$$\begin{gathered} V_0(\alpha) = \frac{V_0(1)}{\alpha}, V_f(\alpha) = \frac{V_f(1)}{\alpha} \\ k_{pt}(\alpha) = \frac{k_{pt}(1)}{\alpha}, k_k(\alpha) = \frac{k_k(1)}{\alpha}, k_{cons}(\alpha) = \frac{k_{cons}(1)}{\alpha} \\ k_{-k}(\alpha) = 1 + \frac{(k_{-k}(1) - 1)}{\alpha} \end{gathered} \tag{23}$$

where $P(\alpha)$ is the notation for the value of parameter P at growth rate α. Parameters λ, K_M and K_N are unaffected by growth rate since these are ratios of concentrations. The effect of changing growth rate on steady state values of P* is shown in Figure S1C. Growth rate (α) was varied from 0.2 to 2 and parameters scaled according to (23). Equation (22) was then used to compute steady state P* values numerically, which were plotted as a function of α. The values of the different parameters at a growth rate of 1 were the same as in Figure S1B, except that $V_0(1) = 0.1$ for the top panel and $V_0(1) = 0.01$ for the bottom panel. At sufficiently low growth rates, the system becomes monostable irrespective of the value of $V_0(1)$ with a steady state P* value that is high.

C. Decoupling of *phoQ (T281R)* expression from PhoP-P regulation

We now consider the steady state behavior of an altered *phoPQ* network where *phoP* expression is still regulated by the native *phoPQ* promoter (and is sensitive to PhoP-P levels), while *phoQ (T281R)* expression is driven by a PhoP-P independent, inducible promoter. In the simple model presented in section A, this decoupling was shown to be sufficient for monostability (cf. equation (11)). An analogous result holds true even when the network is analyzed in detail provided the dissociation constant of the P*Q complex is very large (see below).

In the altered network, equations (14)-(19) still hold. However, one can no longer assume that $Q_{tot} \ll P_{tot}$. Therefore, equation (20) has to be modified to

$$P_{tot} = P + P^* + C^{PQ^*} + C^{P^*Q} \qquad (24)$$

Since Q_{tot} is now a free parameter, equations (14)-(19) along with equation (24) define 7 constraints in 7 variables (6 species, P_{tot}). These can be used to obtain a quintic equation in P* whose roots represent potential steady states of the system:

$$\sum_{i=0}^{5} b_i \,(P^*)^i = 0 \qquad (25)$$

Depending on parameter values, equation (25) can either have 2 or 4 positive real roots (one root is always negative provided $V_0 \neq 0$), which correspond to monostable and bistable regimes. A systematic exploration of parameters was performed to determine the bistable regime (Figure S9). In Figure S9, we can see that bistability is contingent on having a high value of the parameter K_M (compare left and right halves of the figure). In contrast to the situation where both *phoP* and *phoQ* are autoregulated, at low K_M, it is possible to saturate the transcriptional feedback of *phoP* expression (i.e. achieve steady state P* values >1) but not have bistability at low values of V_0. Bistability in the decoupled network is a result of trapping of P* through the formation of dead-end complex P*Q analogous to the stoichiometric sequestration mechanism described in reference [4]. Indeed, even in the monostable regime, the steady-state P* value can be a non-monotonic function of Q_{tot} – at sufficiently high levels of Q_{tot} , further increases in Q_{tot} result in a decrease in steady state P*. This is evident in the right half of Figure S9, which plots steady-state values at high K_M (low dissociation constant of P*Q).

This dependence of bistability on the dissociation constant of P*Q is also depicted in Figure S1D. The following parameters were used to plot Figure S1D: $\lambda = 0.02, V_0 = 0.1, V_f = 20, K_N = 0.1, k_{pt} = 100, k_k = 100, k_{-k} = 10$, and $k_{cons} = 1$. In addition, $K_M = 0.1$ for the left panel and $K_M = 80$ for the right panel. Steady state P* values for these parameters were computed numerically using equation (25) at different values of Q_{tot} and plotted as a function of Q_{tot}.

The monostability of the system in the limit of infinitely large dissociation constant for P*Q can be demonstrated analytically. This limit can be mathematically written as $K_d^{P^*Q} \to \infty$, or equivalently $K_M \to 0$. Substituting $K_M = 0$ in equation (25) simplifies it to a cubic equation:

$$f(P^*) \equiv \sum_{i=0}^{3} c_i \,(P^*)^i = 0 \qquad (26)$$

Moreover, both $c_0 > 0$ and $c_3 > 0$ provided $V_0 \neq 0$. Thus, $f(0) = c_0 > 0$ and $f(P^*) \to -\infty$ as $P^* \to -\infty$. This means that equation (26) has one negative root provided $V_0 \neq 0$. In other words, there cannot be three non-negative solutions to equation (26) and the system has only one non-negative, stable state, i.e., it is monostable.

D. Stochastic simulations of autoregulated and decoupled *phoQ (T281R)* networks

We essentially follow the approach of Kierzek *et. al.* [5] for our stochastic simulations except that we make a fixed volume approximation. We perform simulations on two networks: (1) the *phoQ (T281R)* mutant network (Figure 1A) and (2) the decoupled *phoQ (T281R)* network (Figure 6A). For brevity, we refer to these as autoregulated and decoupled *phoQ (T281R)* networks respectively. The difference between autoregulated and decoupled networks is that in the former, transcription of both *phoP* and *phoQ* is positively regulated by PhoP-P, whereas in the latter only *phoP* is autoregulated by PhoP-P and *phoQ* is constitutively expressed (although the transcription level can be set by an external inducer).

The autoregulated model consists of 7 molecular species and 18 reactions (Table S5), whereas the decoupled model has 8 species and 20 reactions (Table S6). Stochastic simulations were performed using Gillespie's algorithm [6].

We first analyzed the autoregulated model in the deterministic limit. It is straightforward to map the stochastic parameters in Table S5 to their corresponding deterministic model presented in section B by collapsing transcription and translation to a single step, and by setting deterministic fluxes equal to probabilistic propensities. We identified four representative sets of parameters corresponding to monostable OFF, bistable, near-bistable and monostable ON regimes (parameter sets 1-4 in Figure S5A) and performed detailed stochastic simulations with these sets.

To explore the phenomenon of hysteresis, we started simulations with parameter sets 1-4 with either OFF or ON cells. The OFF and ON initial conditions are shown in Table S5. Note that the ON condition approximates the ON stable state computed using deterministic analysis for parameter set 2. For parameter set 1 (monostable OFF regime), we see that irrespective of the starting condition, all simulated trajectories end with very few PhoP-P molecules (Figure S5B, column 1). Parameter set 2 (bistable regime) shows hysteresis that would be expected using deterministic analysis. Both OFF and ON cells mostly retain their state (Figure S5B, column 2). There are, however, some instances of stochastic switching especially in the ON→OFF direction. Such switching events are responsible for the metastability of the ON state demonstrated in Figure 5. With parameter set 3 (near bistable), we see that ON cells remain ON as expected from the deterministic analysis (Figure S5A, column 3). However, a substantial fraction of OFF cells also remained OFF in contrast to deterministic predictions. This is characteristic of the phenomenon of noise-induced bistability [5,7,8]. Far away from the bistable regime (Parameter set 4, Figure S5A, column 4), both OFF and ON cells turn ON as expected from deterministic analysis.

Next, we examined the impact of growth rate and the constitutive promoter (P_2) activity on priming. Starting with parameter set 2 (bistable regime), we performed stochastic simulations with either the normal growth rate, or growth rate decreased by a factor of 2 starting from the OFF initial condition (Figure S5C, columns 1 and 2). Consistent with our deterministic analysis and our experimental results, we find that the reduction in growth rate increased priming from ~0 to ~100%. Thence, we

examined the role of constitutive promoter activity. At the normal growth rate, deletion of the P_2 promoter (modeled by a reduction in constitutive activity by a factor of 10) reduced the number of PhoP-P molecules to almost zero. More importantly, even at the slower growth rate, only ~40% of the cells primed to the OFF state. This is similar to the experimental results documented in Figure 6D.

Finally, we examined the decoupled model. The data presented here corresponds to parameters that are essentially the same as parameter set 2 (bistable regime) for the autoregulated model, except that *phoQ* is no longer regulated by PhoP-P, and is instead expressed at a constitutive (but tunable) rate C_Q (Table S6). As in Figure 6B, we start our simulations with either uninduced or fully induced cells (Table S6, the fully induced starting condition approximates the deterministic steady state for $C_Q = 20$) and then examined the effect of varying the C_Q value. Consistent with the monostability, we find that both starting conditions converge to similar distributions, and that the response is no longer bimodal (Figure S5D). In fact, the mean of the distribution can be tuned by changing the parameter C_Q (Figure S5D). Note that Figure S5D is qualitatively similar to the experimental data presented in Figure 6B.

Overall, we find that the stochastic analysis presented in this section agrees largely with the deterministic analysis in the preceding sections. The main exception is that near the bistable region, one can see the phenomenon of noise-induced bimodality with OFF cells taking a long time to turn ON (Figure S5B, column 3).

References

1. Miyashiro T, Goulian M (2008) High stimulus unmasks positive feedback in an autoregulated bacterial signaling circuit. Proceedings of the National Academy of Sciences of the United States of America 105: 17457-17462.
2. Miyashiro T, Goulian M (2007) Stimulus-dependent differential regulation in the Escherichia coli PhoQ-PhoP system. Proceedings of the National Academy of Sciences of the United States of America 104: 16305-16310.
3. Igoshin OA, Alves R, Savageau MA (2008) Hysteretic and graded responses in bacterial two-component signal transduction. Molecular Microbiology 68: 1196-1215.
4. Tiwari A, Ray JC, Narula J, Igoshin OA (2011) Bistable responses in bacterial genetic networks: designs and dynamical consequences. Math Biosci 231: 76-89.
5. Kierzek AM, Zhou L, Wanner BL (2010) Stochastic kinetic model of two component system signalling reveals all-or-none, graded and mixed mode stochastic switching responses. Mol Biosyst 6: 531-542.
6. Gillespie DT (1976) General Method for Numerically Simulating Stochastic Time Evolution of Coupled Chemical-Reactions. J Comput Phys 22: 403-434.
7. Hermsen R, Erickson DW, Hwa T (2011) Speed, Sensitivity, and Bistability in Auto-activating Signaling Circuits. Plos Computational Biology 7.
8. Hoyle RB, Avitabile D, Kierzek AM (2012) Equation-free analysis of two-component system signalling model reveals the emergence of co-existing phenotypes in the absence of multistationarity. PLoS Comput Biol 8: e1002396.
