# Supplementary material for: The Architecture of a Prototypical Bacterial Signaling Circuit Enables a Single Point Mutation to Confer Novel Network Properties
Source: PLoS Genet. 2013 Aug 22;9(8):e1003706. doi: 10.1371/journal.pgen.1003706 (PMC3750022; doi:10.1371/journal.pgen.1003706)
Supplement: Text S2 — Details of strains and strain constructions. (PDF) [file pgen.1003706.s017.pdf]

Text S2. Details of Strains and Strain Constructions

Tables S1 and S2 list the strains and plasmids used in this study. Table S3 lists primers used for strain construction. A figure-wise list of strains used for collecting data is presented in Table S4. All genetic manipulations were performed using standard protocols [1].

A. Construction of *phoQ (T281R)* Strains (SRI058, SRI066 and SRI078)

SRI058 and SRI066 are the *phoQ (T281R)* strains with and without fluorescent reporters respectively. SRI058 is depicted in Figure 1A. SRI058 and SRI066 were constructed by a two-step scarless mutagenesis procedure described in ref. [2]. In the first step, lambda-Red mediated recombination as described in ref. [3] was used to replace *phoQ* with *cat*-<IsceI> (<IsceI> is the recognition site for the endonuclease I-SceI). A PCR product obtained with primers P1 and P2 and with pWRG100 as template was electroporated into MG1655/pKD46 and chloramphenicol-resistant recombinants were isolated. The resulting strain was named SRI054 and a version of SRI054 with fluorescent reporters (named SRI055) was made by P1vir transduction of SRI054 lysate into TIM92 and selection on chloramphenicol. In the second step, the *cat*-<IsceI> cassette was replaced by *phoQ (T281R)* using the counter-selection protocol described in ref. [2]. A PCR product obtained with primers P3 and P4 and with pTM177 as template was electroporated into SRI055/pWRG99 and SRI054/pWRG99 and counter-selection on LB+50μg/ml ampicilin+500ng/ml anhydrotetracycline plates yielded strains SRI058 and SRI066 respectively.

SRI078 is the chloramphenicol-resistant variant of SRI058. This strain was constructed by P1vir transduction of *cat* from TIM61 into SRI058.

B. Construction of *phoQ (T281R)* ΔP_2 (SRI073)

SRI073 is the derivative of SRI058 with the constitutive promoter (P_2) of the *phoPQ* operon deleted (Figure 6C). This strain was constructed in two steps. In the first step, lambda-Red mediated recombination as described in ref. [3] was used to delete the P_2 promoter in SRI066. A PCR product obtained with primers P5 and P6 and with pKD4 as template was electroporated into SRI066/pKD46 and chloramphenicol-resistant recombinants were isolated. The resulting strain was named SRI069. SRI073 was made by P1vir transduction of SRI069 lysate into SRI058 and selection on chloramphenicol.

C. Construction of Decoupled *phoQ (T281R)* Strain (SRI046)

SRI046 is the strain in which *phoP* is at its native locus and driven by its native *phoPQ* promoter, and *phoQ (T281R)* is relocated to the ϕ_{80} attachment site and is under the control of the IPTG-inducible P_{trc} promoter (Figure 6A). The strain was constructed as follows:

1. Strain SRI030 was constructed by P1vir transduction of FRT-*kan*-FRT cassette from JW1115 (Δ*phoQ*::*kan* from the Keio collection [4]) into TIM210. Strain SRI038 was generated from SRI030

by removal of the *kan* cassette by expression of FLP recombinase using pCP20 [5] and subsequent curing of the plasmid as described in ref. [3].

2. Plasmid pTM168 was integrated at the ϕ_{80} attachment site of TIM13 as described in ref. [6] to generate strain SRI027. Then, lambda-Red mediated recombination as described in ref. [3] was used to delete *phoP* (located at the ϕ_{80} attachment site as part of pTM168) in SRI027 and replace it with a FRT-*kan*-FRT cassette. This was achieved by electroporating a PCR product obtained with primers P7 and P8 and with pKD13 as template into SRI027/pKD46 and selecting for Kanamycin-resistant recombinants. The resulting strain was named SRI035. Strain SRI040 was generated from SRI035 by removal of the *kan* cassette by expression of FLP recombinase using pCP20 [5] and subsequent curing of the plasmid as described in ref. [3]. The CRIM plasmid integrated at the ϕ_{80} attachment site of SRI040 was excised and recovered as described in ref. [6] by transducing a P1vir lysate of SRI040 into BW23473/pAH129 and selecting for chloramphenicol-resistance. The recovered plasmid was named pSR014.

3. Plasmid pSR014 (from Step 2) was integrated at the ϕ_{80} attachment site of SRI038 (from Step 1) as described in ref. [6]. The resulting strain was named SRI046.

D. Construction of ΔP_2 and *phoPQ* Reporter Strains (SRI059, SRI060)

SRI059 and SRI060 are the ΔP_2 Reporter and *phoPQ* Reporter strains referred to in Figure S6. These were constructed as follows:

1. TIM147 has a *yfp* reporter driven by the *phoPQ* promoter integrated at the lambda attachment site. There is also a *cat* gene upstream of the *phoPQ* reporter in this strain. Lambda-Red mediated recombination as described in ref. [3] was used to delete the *cat* gene and the constitutive promoter (P_2) of the P_{phoPQ}-*yfp* reporter in TIM147 and replace it with a FRT-*kan*-FRT cassette. This was achieved by electroporating a PCR product obtained with primers P6 and P9 and with pKD4 as template into TIM147/pKD46 and selecting for Kanamycin-resistant (and Chloramphenicol-sensitive) recombinants. The resulting strain was named SRI043. Strain SRI049 was constructed by P1vir transduction of *kan* from SRI043 into TIM176.

2. In a related construct, the *cat* gene upstream of the *phoPQ* reporter in TIM147 was replaced by a FRT-*kan*-FRT cassette, while keeping the P_2 promoter of the reporter intact using lambda-Red mediated recombination as described in ref. [3]. A PCR product obtained with primers P9 and P10 and with pKD4 as template was electroporated into TIM147/pKD46 and Kanamycin-resistant (and Chloramphenicol-sensitive) recombinants were selected. The resulting strain was named SRI056. Strain SRI057 was constructed by P1vir transduction of *kan* from SRI056 into TIM176.

3. Plasmid pTM168 was integrated at the ϕ_{80} attachment site of SRI049 (from Step 1) and SRI057 (from Step 2) as described in ref. [6] to generate SRI059 and SRI060 respectively.

References

1. Miller JH (1992) A short course in bacterial genetics : a laboratory manual and handbook for *Escherichia coli* and related bacteria. Plainview, N.Y.: Cold Spring Harbor Laboratory Press. 456 p.
2. Blank K, Hensel M, Gerlach RG (2011) Rapid and highly efficient method for scarless mutagenesis within the Salmonella enterica chromosome. PLoS One 6: e15763.
3. Datsenko KA, Wanner BL (2000) One-step inactivation of chromosomal genes in Escherichia coli K-12 using PCR products. Proc Natl Acad Sci U S A 97: 6640-6645.
4. Baba T, Ara T, Hasegawa M, Takai Y, Okumura Y, et al. (2006) Construction of Escherichia coli K-12 in-frame, single-gene knockout mutants: the Keio collection. Mol Syst Biol 2: 2006 0008.
5. Cherepanov PP, Wackernagel W (1995) Gene disruption in Escherichia coli: TcR and KmR cassettes with the option of Flp-catalyzed excision of the antibiotic-resistance determinant. Gene 158: 9-14.
6. Haldimann A, Wanner BL (2001) Conditional-replication, integration, excision, and retrieval plasmid-host systems for gene structure-function studies of bacteria. J Bacteriol 183: 6384-6393.
